# Protein Complexes in Bacteria

**DOI:** 10.1371/journal.pcbi.1004107

**Published:** 2015-02-27

**Authors:** J. Harry Caufield, Marco Abreu, Christopher Wimble, Peter Uetz

**Affiliations:** Center for the Study of Biological Complexity, Virginia Commonwealth University, Richmond, Virginia, United States of America; University College London, United Kingdom

## Abstract

Large-scale analyses of protein complexes have recently become available for *Escherichia coli* and *Mycoplasma pneumoniae*, yielding 443 and 116 heteromultimeric soluble protein complexes, respectively. We have coupled the results of these mass spectrometry-characterized protein complexes with the 285 “gold standard” protein complexes identified by EcoCyc. A comparison with databases of gene orthology, conservation, and essentiality identified proteins conserved or lost in complexes of other species. For instance, of 285 “gold standard” protein complexes in *E. coli*, less than 10% are fully conserved among a set of 7 distantly-related bacterial “model” species. Complex conservation follows one of three models: well-conserved complexes, complexes with a conserved core, and complexes with partial conservation but no conserved core. Expanding the comparison to 894 distinct bacterial genomes illustrates fractional conservation and the limits of co-conservation among components of protein complexes: just 14 out of 285 model protein complexes are perfectly conserved across 95% of the genomes used, yet we predict more than 180 may be partially conserved across at least half of the genomes. No clear relationship between gene essentiality and protein complex conservation is observed, as even poorly conserved complexes contain a significant number of essential proteins. Finally, we identify 183 complexes containing well-conserved components and uncharacterized proteins which will be interesting targets for future experimental studies.

## Author Summary

Though more than 20,000 binary protein-protein interactions have been published for a few well-studied bacterial species, the results rarely capture the full extent to which proteins take part in complexes. Here, we use experimentally-observed protein complexes from *E*. *coli* or *Mycoplasma pneumoniae*, as well as gene orthology, to predict protein complexes across many species of bacteria. Surprisingly, the majority of protein complexes is not conserved, demonstrating an unexpected evolutionary flexibility. We also observe broader trends within protein complex conservation, especially in genome-reduced species with minimal sets of protein complexes.

## Introduction

Abundant genome sequencing revealed an astounding diversity among bacterial genomes. Even species that inhabit the same environment may only share a fraction of their genes. This raises the question how these organisms have adapted to their environments using only a limited number of genes. Here, we investigate the protein complements across bacterial genomes, how proteins are combined into protein complexes across species, and whether these complexes have been conserved across diverse branches on the prokaryotic tree of life.

Other studies have compared the interaction networks of *S*. *cerevisiae*, *S*. *pombe* and *E*. *coli* made possible by systematic screens of genetic interactions and have found notable differences in their structure and content [1,2]. Studies comparing baker’s yeast and fission yeast found that essentiality also varies between species [3]. This might be explained by functional redundancy and the importance of mechanism over structure. The extent of the differences might be unexpected but make sense when seen in the light of evolutionary flexibility [1].

**Numerous studies of protein-protein interactions** have revealed the organization of proteomes into networks of interactions as well as protein complexes. Systematic surveys of protein complexes exist for only a few bacterial species, namely *E*. *coli* [4,5] and *Mycoplasma pneumoniae* [6]. The list of binary protein-protein interactomes is clearly larger but has not been considered in this study. Based on this limited dataset, we investigated whether the complexes found in a few model organisms are sufficient to reconstruct **homologous protein complexes in** other species. This is a particular challenge in prokaryotes as the genomes of most species are highly divergent from the few model species used here. However, *E*. *coli* and *Mycoplasma* provide two important paradigms: *E*. *coli* is a generalist that can live under a variety of conditions while *Mycoplasma* is a specialized parasite that requires host cells to grow. With ~4,300 and ~700 genes, respectively, they represent **medium-sized as well as minimal genomes** and thus medium and minimal diversity of protein complexes.

Few studies have investigated the evolution and diversity of protein complexes across a wide range of taxa. This is not surprising given that large-scale experimental data has only become available in recent years. In combination with a large number of completed genome sequences we can use this data to evaluate **the extent to which protein complexes are likely to be conserved across microbial species**. Furthermore, we can evaluate the biological role of proteins and complexes of unknown function across many species.

Existing studies **comparing sets of interactome data**, including pure bioinformatics approaches [7] have generally limited their comparisons to a few well-characterized protein-protein interaction networks, such as comparisons of *S*. *cerevisiae*, *S*. *pombe* and *E*. *coli* [2,7,8]. Methodological frameworks for predicting co-evolution on the basis of gene presence/absence [1,9] may also be employed to predict novel interactions in other species. In this study, we focus on eight distinct bacterial species, seven of which have been the subject of essentiality screens and two of which have comprehensive protein complex surveys available. We then expand the focus to a set of 894 bacterial genomes.

In order to compare genomes and protein complexes across species, we couple the results of mass spectrometry-characterized protein complexes [5,6] with databases of gene orthology [10] and essentiality [11] to characterize interaction conservation within protein complexes. Furthermore, we use the perspective of genome reduction to evaluate patterns across levels of protein conservation. Comparing sets of protein complexes from divergent bacterial species (in this case, *E*. *coli* and *M*. *pneumoniae*) alleviates some of the bias inherent in using a single species as a universal model. Rather, observing which protein complexes and their components are present in two otherwise distinct species allows us to draw conclusions about how critical these components are to microbial life.

## Results

### Conservation of proteins across bacterial genomes

**Fig 1:**
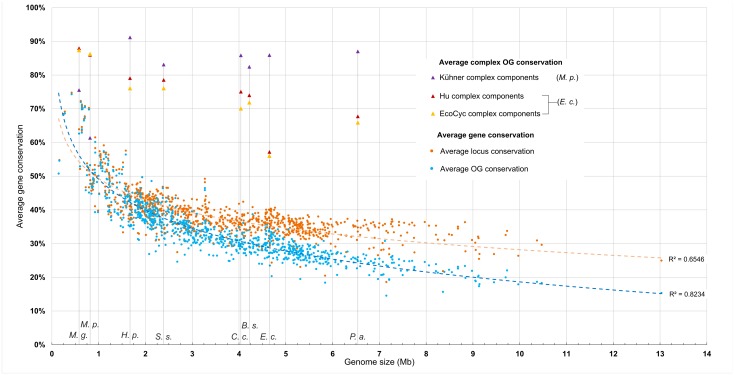
Protein complexes are enriched for highly conserved components. Each point indicates a single genome and the average conservation of its loci or orthologous groups (OGs) as measured by its presence across 898 bacterial genomes. Representative genomes of the 8 species focused on in this study are indicated with vertical lines and the following labels: *M*. *g*., *Mycoplasma genitalium*; *M*. *p*., *Mycoplasma pneumoniae; H*. *p*., *Helicobacter pylori; S*. *s*., *Streptococcus sanguinis; C*. *c*., *Caulobacter crescentus; B*. *s*., *Bacillus subtilis; E*. *c*., *E*. *coli; P*. *a*., *Pseudomonas aeruginosa*. See Materials and Methods for specific genome identities. Average gene conservation is specified as a percentage. Average gene conservation values are reduced by the fraction of their predicted protein-coding genes not present in eggNOG v.3 to account for genes without predicted orthology. To produce OG conservation instead of locus conservation, all but one locus of a set of potential paralogs (in this case, genes sharing the same OG) was removed prior to calculating averages. A logarithmic regression is fitted to both sets of values. Average OG conservation values are also shown for subsets of protein-coding genes present within protein complexes from *E*. *coli* [5] and *M*. *pneumoniae* [6]. For these two species, values are representative of members in full complexomes while those for other species are predicted complexomes using each of the three data sets as models.

In general, small microbial genomes are enriched for proteins which are conserved across bacteria (Fig. 1; Table 1). This trend is most noticeable when paralogy is eliminated, either by removing all but one in a paralogous group (PG) or by the natural effects of genome reduction, as is seen in many of the smallest bacterial genomes. In both cases, average protein conservation decreases as genome size increases. Genes of larger genomes, such as that of *Pseudomonas aeruginosa*, may be conserved across 20 to 30 percent of all other bacteria, on average. The most minimal genomes, including those of *Mycoplasma* species, may share their orthologous groups (OGs) with 60 to 80 percent of other bacterial species, on average. These results are reasonable and expected: reduced genomes, by definition, have lost sequence space but have not lost the loci most crucial to bacterial life itself. Furthermore, though most genomes show an increasing fraction of paralogs being conserved as their size increases (Fig. 1), many of the most reduced genomes actually show greater average conservation when potential paralogs are removed. The paralogous protein-coding loci in reduced genomes may be enriched for accessory genes rather than broadly-conserved core genes.

**Table 1:** Protein conservation across 8 species.

	M. pneumoniae	M. genitalium	B. subtilis	S. sanguinis	H. pylori	C. crescentus	P. aeruginosa	E. coli
M. pneumoniae	**601**	517 (86.02%)	430 (71.55%)	427 (71.05%)	336 (55.91%)	379 (63.06%)	395 (65.72%)	408 (67.89%)
M. genitalium	466 (96.68%)	**482**	388 (80.50%)	381 (79.05%)	290 (60.17%)	333 (69.09%)	353 (73.23%)	355 (73.65%)
B. subtilis	644 (15.88%)	627 (15.46%)	**4056**	1974 (48.67%)	1374 (33.88%)	2101 (51.80%)	2465 (60.77%)	2428 (59.86%)
S. sanguinis	538 (26.37%)	519 (25.44%)	1514 (74.22%)	**2040**	804 (39.41%)	1186 (58.14%)	1341 (65.74%)	1379 (67.60%)
H. pylori	314 (21.43%)	304 (20.75%)	844 (57.61%)	631 (41.21%)	**1465**	916 (62.53%)	979 (66.83%)	992 (67.71%)
C. crescentus	454 (12.58%)	440 (12.20%)	2064 (57.21%)	1487 (41.21%)	1401 (38.83%)	**3608**	2618 (72.56%)	2414 (66.91%)
P. aeruginosa	678 (11.74%)	655 (11.34%)	3275 (56.72%)	2353 (40.75%)	1963 (34.00%)	3615 (62.61%)	**5774**	4007 (69.40%)
E. coli	594 (14.33%)	567 (13.68%)	2418 (58.34%)	1791 (43.21%)	1414 (34.11%)	2409 (58.12%)	3027 (73.03%)	**4145**

On the leftmost column is the organism that is the basis for the comparison while the top row is the organism that is being compared to. An organism compared to itself shows the total number of proteins for that organism in the dataset used. For example, *M*. *pneumoniae* shares 86.02% of its proteins with *M*. *genitalium* while *M*. *genitalium* shares 96.68% of its proteins with *M*. *pneumoniae*. Data for each of the eight species was collected from Uniprot and proteins were mapped to each other using common COGs, NOGs, or bactNOGs [10]

The presence of multiple members within a single orthologous group has an effect on average gene conservation. Here, we display this effect as the difference between average locus conservation and average OG conservation. (Fig. 1, orange vs. blue dots). The difference between the values is an approximation of the level of paralogy across each genome; larger genomes appear to contain more paralogs than smaller genomes, especially as genome size falls below 1 Mb. The effect on average gene conservation is expected, as using orthology-based comparisons compresses paralogs into single OGs. Within our data set, 20 genomes (within 10 unique genera) under 3 Mb had greater average conservation among OGs than among individually-considered loci. The smallest genome in the set, that of the cicada endosymbiont *Hodgkinia cicadicola* [12] demonstrates no difference at all in average conservation between OGs and individual loci. All genomes greater than 3 Mb had higher average conservation among individually-considered loci than among OGs.

### The protein complexomes of *E*. *coli* and *Mycoplasma pneumoniae*

**Fig 2:**
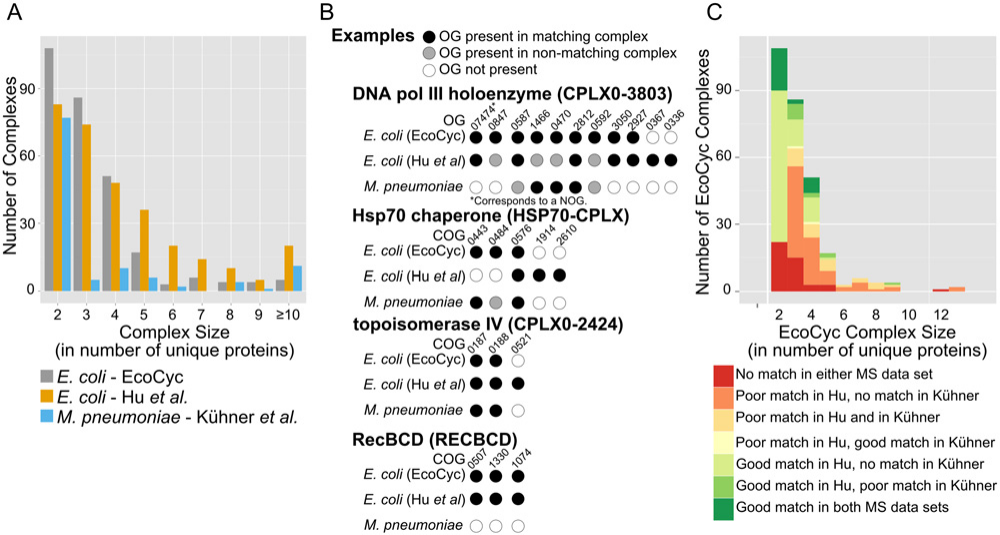
Protein complex data sets vary in composition. **(A)** Count of complexes in two *E*. *coli* complex datasets ([5]; EcoCyc [13]) and one *M*. *pneumoniae* dataset [6], by size (in number of unique protein components). Multimers of single proteins (i.e., homodimers) are not included. **(B)** Examples of complex matching across data sets. Once mapped to an orthologous group (OG), the components of a complex are directly comparable to those in other complex sets yet perfect matches are rare. In some instances, an OG in one complex may not be present in its best matching complex but the OG may be present elsewhere in a different complex. In other cases, the matched complex may contain components (OGs) not seen in the query complex (as is the case with topoisomerase IV). **(C)** Summary of matching complex quality across data sets. EcoCyc complexes were used as the set of query complexes while the two experimental data sets were used as the search space. Here, a poor match requires just one matching component, while a good match requires at least half of the components in the query complex to be present in the matching complex. The number of complexes in each category is shown; complex size is as in part A.

In this study, we used the literature-curated set of EcoCyc *E*. *coli* protein complexes and the protein complexes isolated by Hu et al. [5] as a set of experimentally-determined complexes for *E*. *coli* (Fig. 2A). The set of experimentally-determined *Mycoplasma pneumoniae* complexes identified by Kühner et al. [6] was also included in the comparison as a distantly-related, minimal set. Though these datasets differ in content and approach, both *E*. *coli* data sets contain about 300 complexes. Most complexes in the EcoCyc set contain from 2 to 4 unique proteins while the Hu set contains a comparatively higher number of complexes (more than 30) containing 5 or more unique protein components (i.e, unique proteins mapping to different orthologous groups). Note that some of the Hu et al. complexes appear to represent subsets of full complexes (i.e., the full ribosome constitutes a single complex in EcoCyc but is represented by several complexes in Hu et al.). Also, the EcoCyc set is partially redundant (i.e., each RNA polymerase holoenzyme is represented as a different protein complex, as are the F_1_ and F_0_ subregions of ATP synthase).

The size of the complexes within the data set produced by Kühner et al. appears to differ in distribution from those characterized by Hu et al. (Fig. 2A). Specifically, most *M*. *pneumoniae* complexes with two or more unique members contain just those two unique proteins. The cross-species discrepancy may also result from methodology, though Kühner et al. suggest it is representative of authentic biological differences between the two species. *M*. *pneumoniae* contains fewer unique proteins than *E*. *coli* does and this difference limits the number of unique proteins seen in any single complex.

The exact protein complexes defined by each data set differ. Pairwise comparison of presence or absence of proteins in each complex is improved by mapping components to orthologous groups but few complexes appear to be present in an identical form across all three data sets. Fig. 2B provides four examples of the types of complex matches seen across the data sets. For instance, the DNA polymerase III holoenzyme (EcoCyc: CPLX0–3803) contains 9 unique proteins as per EcoCyc but its closest match in the Hu set contains 7, including two proteins not found in any EcoCyc complex. The “missing” proteins from the EcoCyc complex are found in other Hu complexes. The Hsp70 chaperone complex (EcoCyc: HSP70-CPLX) provides another example: The *M*. *pneumoniae* complexes provide a better match for the EcoCyc complex than the Hu set does. Topoisomerase IV (EcoCyc: CPLX0–2424) has a good match in all three data sets though the representative Hu complex contains an additional protein. Lastly, RecBCD serves as an example of a good *E*. *coli-*specific match with no components present among the *M*. *pneumoniae* complexes.

In the aggregate, most EcoCyc complexes do not have reliable matches in the other experimental sets (Fig. 2C). Using all 285 EcoCyc complexes as a guide, their best matches in the other sets are classified as “good” if they contain at least half of the same unique proteins (as members of orthologous groups) or “poor” if they contain a match of less than half of the EcoCyc complex’s components. No complex of a size greater than 4 unique proteins has a good match in both the Hu et al. and Kühner et al. complex sets. 28 complexes (9.8%) of the complexes of size 4 or less have good matches in both sets. The majority of the complexes in this size class (153 out of 246) contained at least one matching component in the Hu *E*. *coli* complexes but no match among the Kühner et al. *M*. *pneumoniae* complexes.

The set of *M*. *pneumoniae* complexes serves as a rough model for the complexes most commonly found across bacterial species (see S9 Table and S10 Table) for the predicted conservation of each complex). It is an imperfect model: out of 116 complexes, only 28 are fully conserved (that is, each of their components are present as orthologs) in the 7 other model species in this study. 39 *M*. *pneumoniae* complexes appear to share at least 2/3 of their components with all the other species, though 75 complexes share at least half. Just one complex contains components entirely specific to *M*. *pneumoniae* (complex 87, composed of uncharacterized proteins Mpn036 and Mpn676, respective UniProt entries P75078 and P75116).

### Using protein complexomes to predict complexes conserved in other species

**Fig 3:**
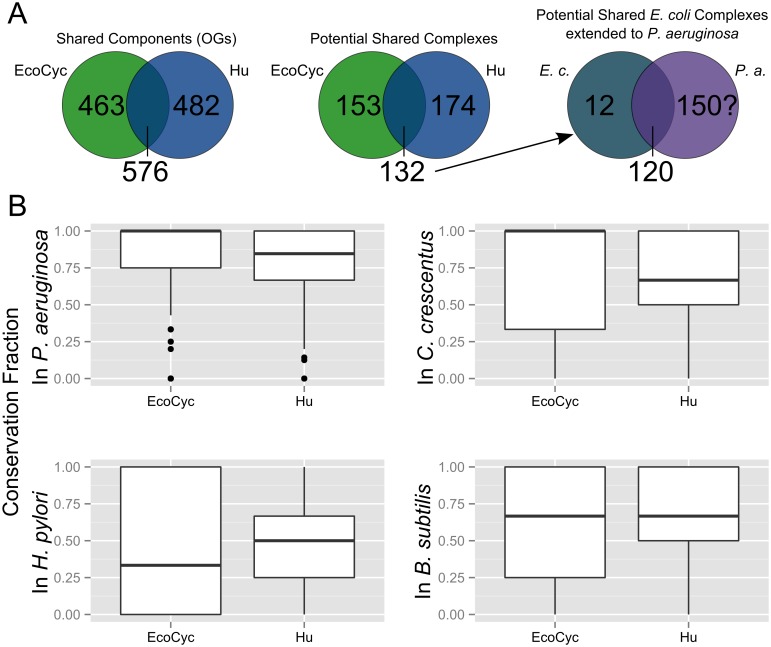
Protein complex sets vary in conservation across bacteria. **(A)** Overlap between literature-curated (EcoCyc) and experimentally-observed (Hu et al.) *E*. *coli* complex sets is limited. Each data set contains unique proteins, even when all are mapped to orthologous groups (far left). Each complex in one of the two *E*. *coli* complex sets may or may not appear to be shared in the other complex set (middle; a potentially shared complex must have at least half of its components in at least one complex in both sets). Using just the set of complexes shared between the two *E*. *coli* sets as a model for predicting complexomes in other species (far right; in this case, *P*. *aeruginosa* is used as an example) may be limiting. 12 complexes from the shared set appear to be conserved in *P*. *aeruginosa* but roughly an additional 150 complexes may be expected based on those seen in *E*. *coli*. **(B)** Each box plot displays the range of conservation fractions of *E*. *coli* protein complexes from the literature curated (EcoCyc) and experimental (Hu et al.) sets with respect to a species other than *E*. *coli*. The upper and lower edges of each box correspond to the first and third quartile of conservation fraction values, respectively. The upper whisker corresponds to the highest value within 1.5 times the inter-quartile range (IQR) while the lower whisker corresponds to the lowest value within the same range. Data points outside 1.5 times IQR are represented by single data points.

The variability between the EcoCyc and Hu datasets has a direct impact on the usefulness of these complexomes as models for other bacterial species. In any case, the EcoCyc and Hu complex sets provide the most comprehensive complex set currently available for *E*. *coli*. The intersection of the two sets (Fig. 3A) is indeed limited: just 576 unique orthologous groups are shared between the sets and just 132 complexes appear to be “good” matches between the sets. Using these 132 complexes as a model for those in *P*. *aeruginosa* shows that up to 120 of the complexes may be conserved based on orthologous components present in the *P*. *aeruginosa* genome. If the yet-uncharacterized *P*. *aeruginosa* complexome contains roughly the same number of complexes as those for *E*. *coli* then this prediction method misses more than half (that is, around 150) of the potential complexes unless we also use the unique complexes of each set. We used these results as evidence that the data sets should be used as independent models rather than as an intersecting set: losing more than half of the potential model complexes simply due to inconsistencies across data sets may be too limiting for a broad cross-species comparision.

Fig. 3B displays distributions of protein complex conservation across four bacterial species other than *E*. *coli*. (*M. pneumoniae* complexes were not used in this comparison.) These plots provide the median and interquartile range of protein complex conservation fractions in each species, using either EcoCyc or Hu et al. complexes as a model of the complex set. A comprehensive set of protein complexes has not been identified for any of these species as of yet. Following the results shown in Fig. 1, however, we may predict that most bacterial protein complex component sets should share at least half of their OGs with all other bacterial genomes, on average. Basic biology also plays a role here: we expect a subset of crucial protein complexes like polymerases to be well-conserved across all species. The set of all EcoCyc complexes, appears to be highly-conserved in *P*. *aeruginosa* (the entire interquartile range lies between full and 75% complex conservation, showing the average EcoCyc complex is well-represented in *P*. *aeruginosa*) but shows a greater range of conservation across the three other species. The Hu complexes show lower complex conservation median values than EcoCyc for all but *H*. *pylori* and lower variability for all but *P*. *aeruginosa*. Here, the median values are not as useful as the conservation ranges: the distance between the highest and lowest values includes every possibility from 0 to 100% conservation using either model of *E*. *coli* complexes. We see that the two species most closely related to *E*. *coli* in this set—*P*. *aeruginosa* and *C*. *crescentus—*produce different median values and interquartile ranges between the sets across all protein complexes. Components of complexes in the two *E*. *coli* sets, used as models, are clearly conserved differently across bacterial species. A higher-resolution comparison is necessary to determine which complexes are highly-conserved.

### Protein complexes and their essentiality are poorly conserved in bacteria

Although the size distribution is different in *E*. *coli* and *Mycoplasma*, we hypothesized that homologous complexes should be very similar, both in size and composition. However, this is not true: few complexes share even half of their components across the data sets (Fig. 2C). The majority of complexes shows less than 50% overlap between EcoCyc and Hu, but also between Hu and *Mycoplasma*. This suggests that there are both technical (*E*. *coli vs E*. *coli*) but also biological reasons (*E*. *coli* vs. *Mycoplasma*) for these differences.

**Fig 4:**
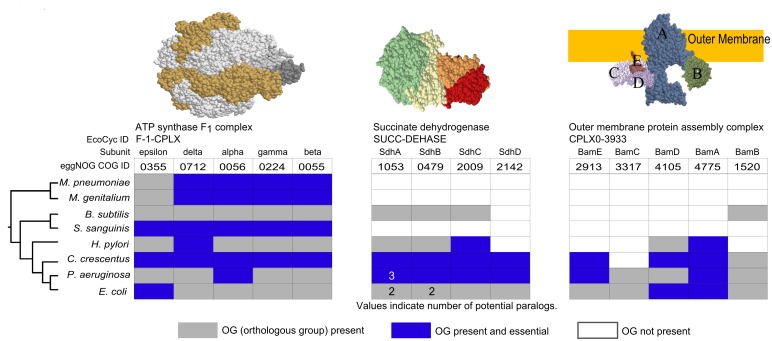
Examples of protein complex conservation across bacteria. Complexes are identified using a common name and an EcoCyc ID. Each complex subunit has been assigned a COG ID. Grey areas indicate OG presence, white areas indicate OG absence, and blue areas indicate essentiality in a species-specific screen (see Materials and Methods for references). Values within these spaces indicate the presence of potential paralogs in the corresponding species; proteins without specified values have no paralogs. Complex structures are available in PDB: ATP synthase F1, 3OAA; succinate dehydrogenase, 1NEN; Outer membrane protein assembly complex, (2KM7, 3TGO, 3TGO, 4K3C, 2YH3). Species are arranged by their taxonomy (see Materials and Methods for details) with *E*. *coli* and *Mycoplasma* serving as the most distant species.

To get a more global yet more detailed picture of protein complex conservation, we compared conservation across 8 bacterial species, including the two species for which full protein complex sets exist. The EcoCyc complex set was used as a standard to which all other species were compared. Fig. 4 provides three examples of the ways protein complexes may or may not be conserved across species. Conservation of protein complexes may be roughly grouped into three categories: well-conserved complexes, complexes with a core set of proteins conserved, and those in which no core set appears to be consistently conserved. As conservation and essentiality may be related to paralogy, we also compared the components of these complexes on the presence or absence of paralogs.

It is commonly assumed that highly conserved proteins must be important and thus should be essential in many cases. Interestingly, this is often not true (Fig. 4). For example, the well-conserved **succinate dehydrogenase** components are essential in only 3 of the species shown. The four components of this complex (as defined by the default structure in *E*. *coli*) are present only in *Pseudomonas aeruginosa* and *Caulobacter crescentus*. *Helicobacter pylori* and *B*. *subtilis* encode 3 out of 4 components and the other 3 species appear to have lost the entire complex. Similarly, the **Bam outer membrane protein assembly complex** (EcoCyc: CPLX0–3933) shows partial essentiality across the complex in 4 species though its components are well conserved in only 3 species. This complex has a similarly patchy pattern of conservation, with any number from zero to all 5 components conserved. In the case of *H*. *pylori* Bam complex, what initially seems like a lack of conservation may be the result of component replacement by functionally similar proteins [14]. By contrast, **F_1_ ATP synthase** is conserved in all species examined. These examples show that most complexes are less well conserved than their often important functions indicate (as measured by the presence of essential proteins in these complexes).

**Fig 5:**
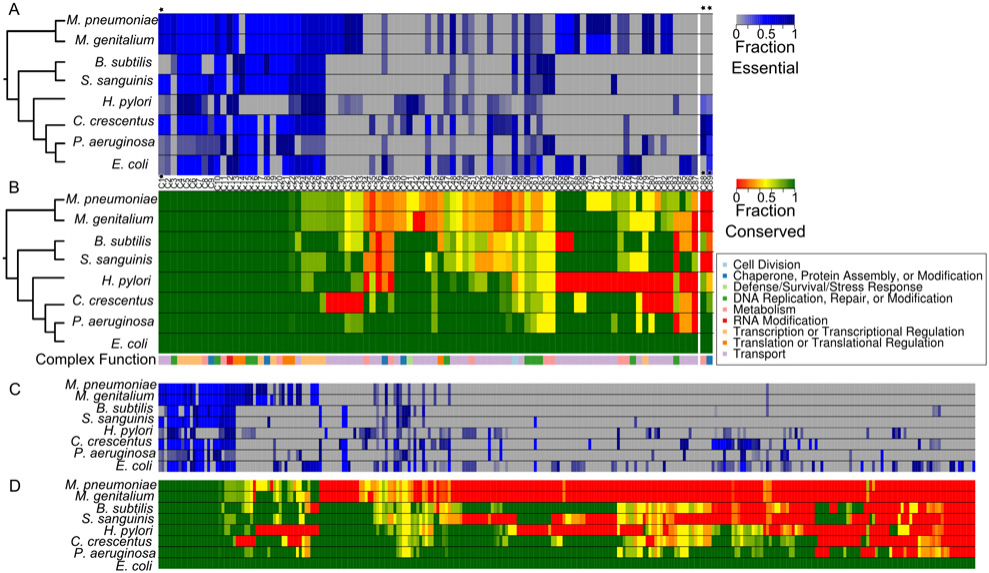
Fractional essentiality and conservation of protein complexes across species. **(A)** Each column represents one protein complex (as defined in EcoCyc for *E*. *coli*) and its fraction of essentiality within the species shown at left. This subset of complexes are those in which at least one component is predicted to be conserved in *M*. *pneumoniae*. Black stars by complex IDs indicate complexes shown in Fig. 3. Two example complexes not predicted to be present in *M*. *pneumoniae* are also shown at the far right of the complex list. See S1 Table for key to complexes. For species other than *E*. *coli*, complexes are predicted using orthologous groups (OGs). Colors indicate the fraction of essentiality: blue—conserved components are essential at the fraction specified at right, grey—no components are conserved or all conserved components are not essential. **(B)** Conservation of complexes as shown in (A). Colors indicate the fraction of conservation ranging from dark green (all proteins are present) to red (no protein is present). General functional group assignments were manually assigned based on EcoCyc annotations. Columns in panels A and B correspond to the same complexes. **(C)** As in part A, but for the full set of EcoCyc *E*. *coli* complexes; each column is a single complex. An extended version of this heat map is provided in S1 Fig. **(D)** As in part B, but for the full set of EcoCyc *E*. *coli* complexes; each column is a single complex. The order of complexes is identical to that in (C). An extended version of this heat map is provided in S2 Fig. Columns in panels C and D correspond to the same complexes.

Fig. 5A displays all EcoCyc *E*. *coli* complexes with at least one component present in *M*. *pneumoniae*. In this case, fraction of essentiality (the number of protein components found to be essential out of all protein components present) is shown. Fig. 5B displays conservation fractions of all *E*. *coli* complexes with at least one protein conserved in *M*. *pneumoniae*, though not necessarily present in a *M*. *pneumoniae* complex. A complete survey of all EcoCyc complexes across these species in terms of conservation and essentiality is provided in S1 Fig. and S2 Fig., respectively. Conservation fraction was established as the fraction of unique proteins in a defined complex present in the target species. Notably, proteins of only 21 complete EcoCyc complexes are fully conserved across all 8 species, or just 15 complexes when subunits and alternate forms (i.e., RNA polymerase with different sigma factors) are removed. An additional 19 complexes are fully conserved across all species but the two *Mycoplasma* species. The remaining complexes vary extensively in their degree and extent of conservation. A number of complexes are well conserved across *E*. *coli*, *P*. *aeruginosa*, *C*., *crescentus*, *H*. *pylori*, and *B*. *subtilis* but not *S*. *sanguinis* or the *Mycoplasma (e*.*g*. succinate dehydrogenase, EcoCyc: SUCC-DEHASE). Overall, of the 176 EcoCyc complexes of 3 or more unique proteins (S1 Fig.), 128 appear to have lost at least one unique protein component in one or more species. This demonstrates that protein complexes are far more flexible in evolutionary terms than previously assumed.

Protein complex function varies in a similar way as conservation (Fig. 5B). As expected, many of the most highly conserved complexes are directly involved in DNA replication, transcription, or translation. Many protein complexes of varying conservation fractions are transport complexes—as bacterial membrane structures vary across species, some degree of transporter component evolution is also expected. At least six distinct complexes involved in DNA modification or repair demonstrate less than perfect conservation.

*E*. *coli* complexes serve as a “gold standard” for protein complexes across bacteria only in cases where most or all of the components of a complex are broadly conserved. This property is true of just a small fraction of complexes. Fig. 5D displays conservation fractions for all 285 *E*. *coli* complexes in the EcoCyc set, clustered by similarity of their conservation patterns across the 7 other species used in this study. Just 21 complexes appear to be fully conserved (that is, orthologs of each of their components are present) in all other species. This is a broad taxonomic range, so a more relaxed cutoff may be appropriate to predict a complex is conserved; even so, only 28 complexes contain at least 2/3 of the *E*. *coli* components across all species. Lowering the cutoff to at least half of the *E*. *coli* components still yields only 34 complexes. The lack of broad conservation is not, however, a matter of full complex presence or absence across species. Rather, many complex components appear to be conserved independently from other members of their complex. Similarly patchy conservation can be seen for essentiality (Fig. 5C), as the most broadly well-conserved complexes (far left) generally retain essentiality across species but less consistently-conserved complexes do not, though they may retain essentiality while appearing to lose complex components.

### The *E*. *coli* protein complexome as a model for other species

**Fig 6:**
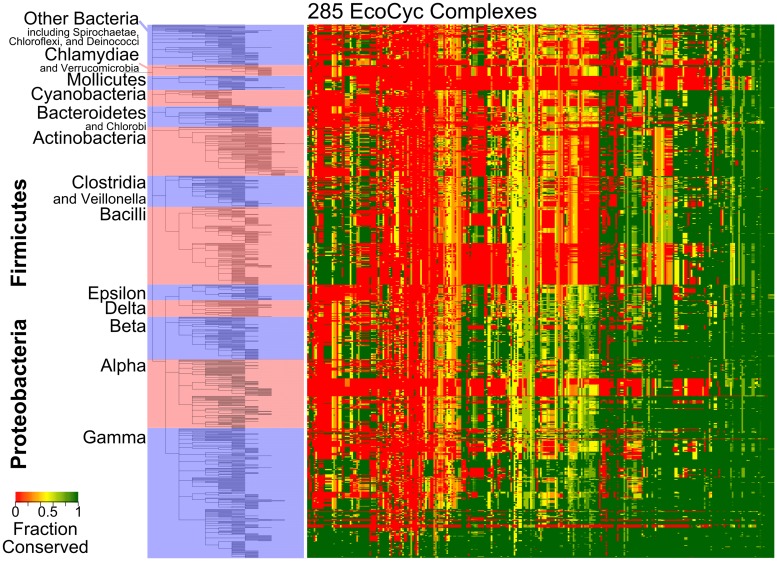
*E*. *coli* complex conservation across Bacteria corresponds to taxonomic boundaries. The heat map displays fractional conservation of all 285 EcoCyc protein complexes as in Fig. 5, though in this case across 894 different bacterial genomes as indicated on the tree at left. See Materials and Methods for taxonomic details. Specific complex names and species/strain names may be found in S5 Fig. Complexes (columns) have been clustered on the basis of the distance between their average fractional conservations (average linkage).

*E*. *coli* is frequently used as a model organism for bacteria in general. Using the literature-curated set of protein complexes from EcoCyc, we sought to determine how well this protein complexome serves as a model for complexes in other bacterial species. A comparison of the fractional conservation of each EcoCyc complex across 894 different bacterial genomes was the result (Fig. 6; see S5 Fig. for an expanded version). The genomes in this comparison were arranged as per NCBI taxonomy definitions, revealing patterns in complex conservation closely corresponding to numerous taxonomic boundaries. Hierarchical clustering of each *E*. *coli* model complex (specifically, UPGMA) on the basis of its fractional conservation across all other species reveals groups of complexes with similar patterns of predicted conservation.

The species with the most overall conservation of the *E*. *coli* complexes are, unsurprisingly, those most closely related to *E*. *coli*. Roughly a third of the complex set is conserved across all species with the minimal *Rickettsia* and *Mycoplasma* genomes, among others, serving as notable exceptions. The middle third shows the most difference in conservation between the Proteobacteria and all other species. The Lactobacillales show the most difference in conservation among these complexes, to the degree that they resemble *Cyanobacteria* more closely among this subset. The last third (far left of Fig. 6) of the complexes demonstrate the most variable conservation across all species. Many of these complexes are missing or partially conserved among the Proteobacteria yet are fully present in many Firmicutes species and even in extremophiles like *Thermus* or *Thermotoga* species. Overall, out of 285 EcoCyc complexes, 12 (~4%) have at least one component present in all 894 bacterial genomes in the set. None are perfectly conserved across all genomes but 14 complexes appear to be conserved across at least 95% of the genomes. If potential complex conservation is generously defined as conservation of at least half of the complex components, 3 EcoCyc complexes are potentially conserved across all 894 genomes, 25 are potentially conserved across 95% of the genomes, and 186 are potentially conserved across at least half of the genomes. Variance across the full set of complex conservation fractions is 0.189. Because conservation of these complexes follows the existing taxonomy well, some generally well-conserved complexes like RNA polymerases may be missing from entire genera.

The experimentally-determined protein complexes identified by Hu et al. were also used as a model of the *E*. *coli* complexome (S6 Fig.). Most complexes appear to have partial conservation across nearly all species using this model. Distinctions are still seen among the minimal genomes of the Rickettsiales as well as the *Mycoplasma* and the genomes of related species. Out of 310 Hu et al. complexes, 16 (~5%) have at least one component present in all 894 bacterial genomes in the set. As with the EcoCyc complexes, none are perfectly conserved across all genomes but a single complex (complex 271) appears to be conserved across at least 95% of the genomes. Using the same 50% cutoff for potential complex conservation as used above, no Hu complexes appear to be conserved in all 894 genomes, 10 are potentially conserved across 95% of the genomes, and 182 are potentially conserved across at least half of the genomes. Though these Hu et al. complex values appear similar to those for the Ecocyc complexes, variance across the full set of Hu complex conservation fractions is 0.097, indicating less variability among the values than that seen for the EcoCyc complexes. This lesser variance can also be seen in the surprising consistency across taxonomic lines (S6 Fig.).

Both the literature-curated EcoCyc model and the Hu et al.-based experimental model were evaluated by comparision to a randomized version of their respective components. For the literature-curated model, Pearson correlation was 0.185, while for the experimental model, Pearson correlation was 0.293. The higher correlation value for the experimental model indicates it is closer to a random distribution of complex correlation fractions across the species set. We do not expect complexes to be conserved in a random pattern so this may indicate the Hu et al. complex set is less useful than the EcoCyc complex set for prediction across this wide range of genomes.

### Essentiality of proteins in complexes and the impact of paralogy

**Fig 7:**
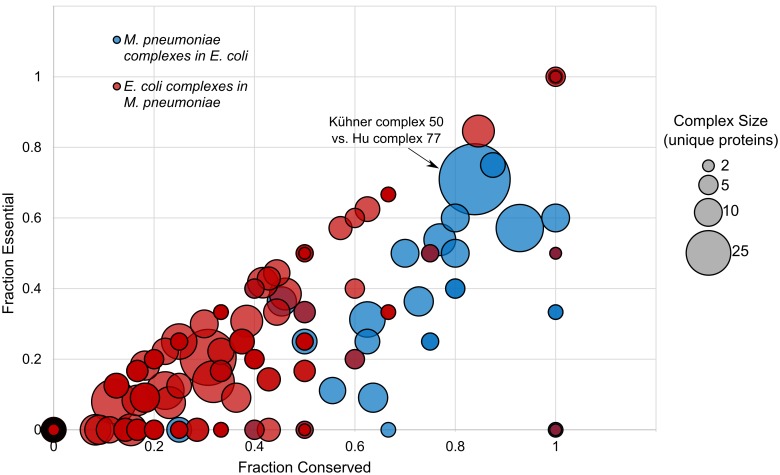
Conserved complex components are enriched for essential proteins. This correlation is even more pronounced in *Mycoplasma* (blue). Protein complexes of *E*. *coli* [5] are compared to complexes of *M*. *pneumoniae* [6] and *vice versa*. Fraction of conservation and fraction of essentiality are calculated as described in Materials and Methods. Each node represents a single protein complex with relative size corresponding to the size of the complex in number of components. Kühner complex 50 and its corresponding Hu complex 77 are indicated as an example complex match.

*Mycoplasma* species have highly reduced genomes and it is generally assumed that they have retained mostly essential proteins. In fact, the fraction of conserved essential proteins is much higher when comparing *Mycoplasma pneumoniae* to *E*. *coli* than vice-versa (Fig. 7). In these comparisons, all complex components are searched for in full genomes and essentiality is assigned based on the target species. Among the full set of Hu et al. *E*. *coli* complexes, complexes have an average conservation fraction of 0.198±0.230 and an average essentiality fraction of 0.122±0.196 in *M*. *pneumoniae*. High variability in conservation among complexes is expected as complex components, like single proteins, are subject to a broad variety of evolutionary pressures. Among the 53% of complexes with at least one component present in *M*. *pneumoniae*, the average fractions increase to 0.375±0.184 and 0.231±0.218, respectively. Among the full set of Kühner et al. *M*. *pneumoniae* complexes, complexes have an average conservation fraction of 0.716±0.292 and an average essentiality fraction of 0.32±0.332 in *E*. *coli*. Among the 95% of complexes with at least one component present in *E*. *coli*, the average fractions increase to 0.755±0.245 and 0.337±0.332, respectively. Overall, *Mycoplasma* protein complex components are more likely to be present and essential in *E*. *coli* than *E*. *coli* protein complex components are in *Mycoplasma*.

One possible explanation for the lower fraction of conserved essential proteins in *E*. *coli* is the presence of paralogs that renders duplicate genes non-essential, given the presence of an additional copy with a redundant function. We performed comparisons of the fraction of conservation of each complex and its **sum of paralogy** (that is, the total number of all copies of all genes coding for the complex components in the target species). As the number of paralogs for each gene was broadly defined using orthologous groups, these numbers are considered maximum possible values rather than specific counts of known paralogous regions. We observed an inverse trend between *E*. *coli* complexes vs. *M*. *pneumoniae* (S3A Fig.) and *vice versa* (S3B Fig.): the more paralogs they have in *E*. *coli* the less conserved these proteins were in *Mycoplasma* and *vice versa*. More specifically, *E*. *coli* complexes with a conservation fraction greater than 0.6 in *M*. *pneumoniae* all had total paralogy sums lower than 40 though more poorly-conserved complexes had paralogy sums between 2 and about 100. *M*. *pneumoniae* complexes with a conservation fraction greater than 0.6 in *E*. *coli* had a range of sums of paralogy between 2 and nearly 80. The more poorly-conserved complexes all had paralogy sums of 60 or less. Calculated Pearson anti-correlation for *E*. *coli* complexes vs. *M*. *pneumoniae* (S3A Fig.) was -0.04 and Pearson correlation for *M*. *pneumoniae* complexes as a model for *E*. *coli* (S3B Fig.) was 0.05, indicating limited to no overall correlation in either full comparison. As is the case with conservation of complexes across all species (Fig. 6), correlation may be case-specific.

**Fig 8:**
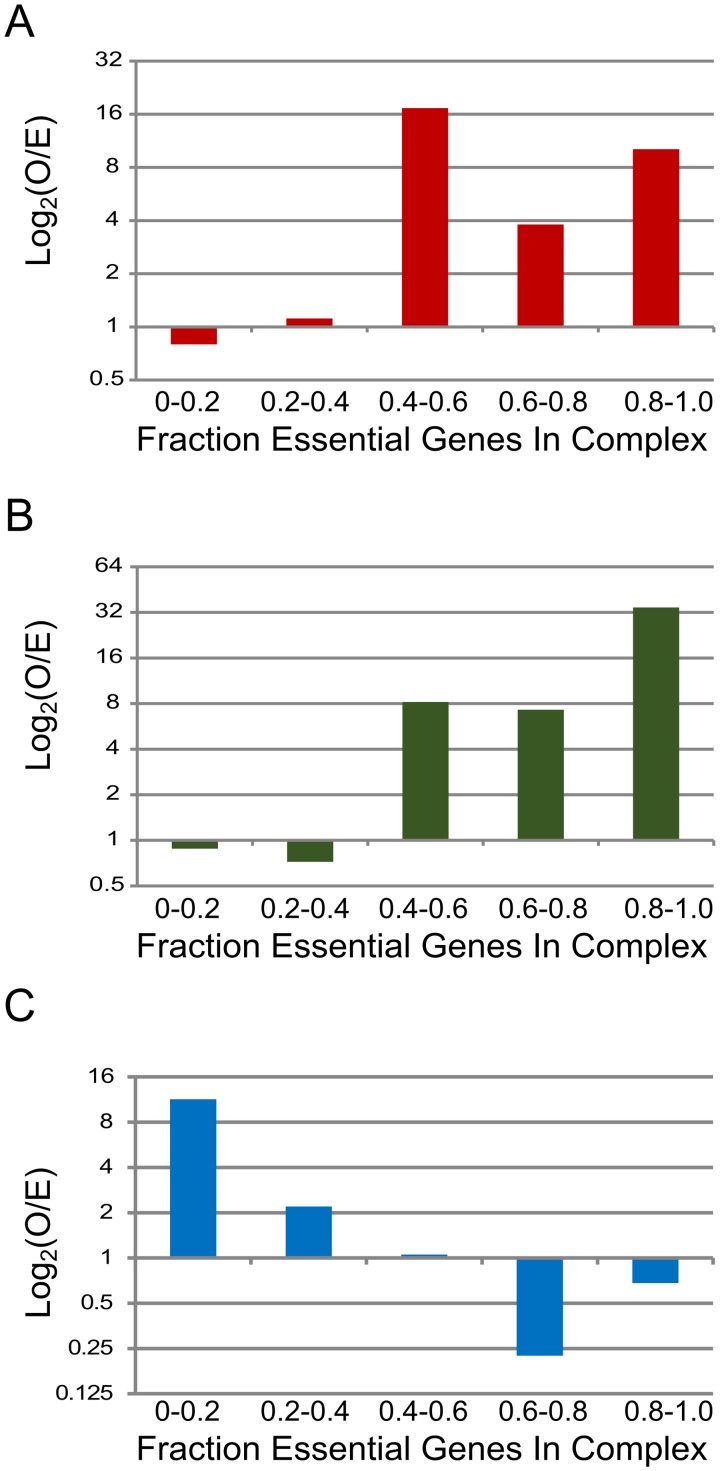
Essentiality of proteins in complexes. Distribution of essential genes among those from *E*. *coli* (Hu et al. **(A)** and EcoCyc **(B)**, respectively) and *M*. *pneumoniae* **(C)**. The fraction of essential genes within protein complexes was determined for each complex set. In *E*. *coli*, essential protein complexes are enriched for essential proteins. By contrast, complexes with non-essential proteins are over-represented in the genome-reduced *Mycoplasma pneumoniae*. Each distribution is expressed as binned log_2_ ratios of observed over expected frequency. Values indicate observed frequency above or below random results (= 1), respectively.

The fraction of essential components in protein complexes is non-random and may be greater than expected, depending upon the complexes compared (Fig. 8). When compared to random assortment, Hu et al. *E*. *coli* complexes have more essential proteins than expected by chance (Fig. 8A). A Spearman anti-correlation of -0.25 was found. *E*. *coli* complexes from EcoCyc (Fig. 8B) demonstrate similar trends, with a Spearman anti-correlation of -0.22. *M*. *pneumoniae* complexes from Kühner et al. (Fig. 8C) show a trend of declining essentiality compared to randomized essentiality fractions of 0.6–08. A Spearman anti-correlation of -0.03 was found for this *M*. *pneumoniae* complex set. Both *E*. *coli* anti-correlations show a weak relationship.

Paralogy was also examined as a function of essentiality (S4 Fig.). Here, average paralogy values were determined for each complex to minimize the impact of complex size, especially as only one or two components of a complex may be essential. *E*. coli complexes from Hu et al. (S4A Fig.) decrease in average paralogy as their fraction of essentiality decreases. In total, compared to random assortment, far more *E*. *coli* complexes than expected appear to have essentiality fractions of 0.4 or more. *E*. *coli* complexes from EcoCyc (S4B Fig.) demonstrate similar trends. *M*. *pneumoniae* complexes from Kühner et al. (S4C Fig.), however, do not appear to retain the same relationship between essentiality and average paralogy. Additionally, more *M*. *pneumoniae* complexes than expected were found to have essentiality fractions of 0.2 or less while fewer than expected had essentiality fractions greater than 0.6. Spearman anti-correlation was not statistically significant at -0.03. Overall, essentiality and average paralogy appear to be related for *E*. *coli* but not for *M*. *pneumoniae* complexes, probably because *M*. *pneumoniae* contains relatively few paralogs. Example complexes from each of these sets and the OGs shared between them are provided in S4D Fig.; in each example, at the majority of the complex components are essential but their representative genomes contain few paralogs coding for redundant complex components.

### Proteins of unknown function

**Fig 9:**
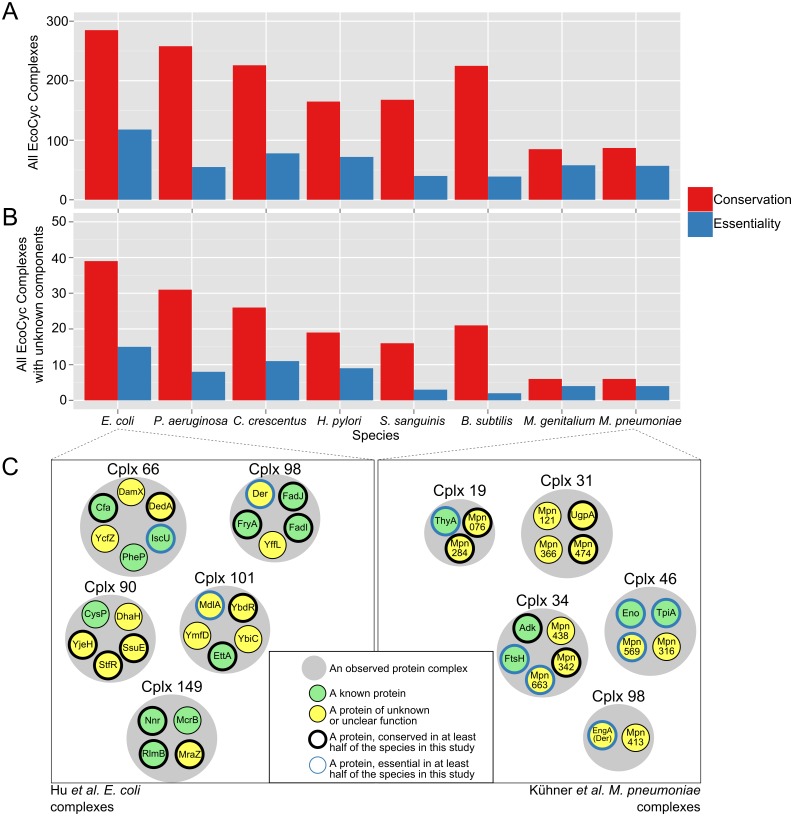
Protein complexes are rich in highly-conserved proteins of unknown function. **(A)** The list of EcoCyc *E*. *coli* protein complexes was compared on the basis of component presence vs. absence across seven other species in this study. Conserved complexes, in this figure, are those in which at least one orthologous component is present in the target species. Similarly, essential complexes include at least one component found to be essential in both *E*. *coli* and in the target species. **(B)** As in (A), but within the subset of EcoCyc complexes containing at least one protein of unknown or unclear function. In these instances, the complex itself may have a known function though the roles of its components may remain unclear. **(C)** Examples of experimentally-observed protein complexes containing proteins of unknown function. *E*. *coli* complex examples from Hu et al. are shown at left, *M*. *pneumoniae* complexes from Kühner et al. are shown at right. Complexes are labeled with the identifier used in their corresponding study.

Protein complexes are attractive targets for functional analysis, given that proteins are embedded in a functional context. This is especially true for proteins of unknown function that are part of a complex (Fig. 9A, B). Here, conservation is defined as greater than 0.5 conservation fraction and essential complexes are those with at least one essential component in the target species. Among the highly conserved components, many are essential in 4 or more of the 8 species. Using more than one species reduces the effect of noise and inconsistency across essentiality screens. Starting with 39 EcoCyc-defined complexes that contain unknown proteins, at least 15 appear to be conserved in all other species in this study but the *Mycoplasma*. Fig. 9C displays example complexes for the Hu (*E*. *coli)* and Kühner *(Mycoplasma pneumoniae)* complexes, respectively. Unlike in parts A and B, the complexes shown are experimental results rather than literature-defined complexes. Each complex contains at least one component of unknown or unclear function, whether in the context of the protein complex or broader cellular function. For instance, complex 66 from Hu et al. (Fig. 9C) consists of 6 unique proteins of which 3 are of unknown function (or remain without annotation). Of the 6 proteins, 3 are highly conserved and 1 of those three is frequently essential. The *E*. *coli* protein MraZ, present in Hu complex 149, is shown here as a protein of unknown function but was recently found to be a transcriptional regulator involved in multiple pathways [15]. More than 149 Hu et al. *E*. *coli* complexes and 34 Kühner et al. *Mycoplasma pneumoniae* (183 in total) complexes contain at least one component of unknown function. Of these, 109 Hu et al. *E*. *coli* complexes and 19 Kühner et al. *M*. *pneumoniae* complexes contain components highly conserved as essential proteins. The full list of experimental complexes with unknown components is available in S11 Table.

## Discussion

The substantial variation among protein complexes across species supports the notion that these complexes are much more malleable than previously thought. A possible explanation of this is that the function of a complex is more important than its content. Complexes can serve the same role yet contain different proteins and when one function is lost, others can fill in the gap. Other studies have found that functional redundancy can lead to variation and that there is little overlap in terms of protein interaction among species [2,3]. While mutational change in a protein complex may have catastrophic potential, complexes are not immutable. In fact, several complexes that are essential in some species have varying composition in other species. For instance, 5 out of 9 components of the *E*. *coli* Sec translocation complex (EcoCyc: SEC-SECRETION-CPLX) are well-conserved across species from *P*. *aeruginosa* to *M*. *genitalium*. One of these components, SecA, has been found to be essential in all species focused on in this work with the exception of *S*. *sanguinis*; orthologs of this protein are present in all 894 bacterial genomes examined. The remaining 4 *E*. *coli* components are more variable in conservation across species. For instance, YajC is present in 727 out of the same 894 genomes. Strong selection pressure seems to avoid mutations that render the entire complex ineffectual. This may explain why we have observed a higher level of conservation for protein complex components than for proteins in general (Fig. 1).

The essential “core” components of protein complexes may be conserved across taxonomic levels while “accessory” components may not [1]. Given their multiple interactions, proteins within protein complexes should not only be more highly conserved than “un-complexed” ones, but should retain their essential roles if their fellow complex members are present [16,17].

Components of protein complexes are, on average, more likely to be present in other bacterial species than proteins not in complexes [1]. This is a result of high conservation among sets of large, essential complexes. 128 out of 285 literature-verified *E*. *coli* protein complexes are fully present in *B*. *subtilis*, 30 of which are also completely present in *M*. *genitalium*. For instance, all components of the ATP synthase complex (EcoCyc: ATPSYN-CPLX) are present in all species examined, though they do vary in essentiality. *B*. *subtilis* essentiality screens found no essential genes in ATP synthase, while those for *M*. *pneumoniae* found all but one component to be essential. Other complexes—predominantly those with transmembrane domains and/or transporter functions—are more variable in both conservation and essentiality, though they provide examples of how dispensable accessory proteins may be.

Some protein complexes with essential functions in *E*. *coli* may not be present in other species. The lipopolysaccharide transport complex (EcoCyc: CPLX0–7992) serves as an excellent example: all seven of the Lpt proteins in this complex have been found to be essential in *E*. *coli* though their conservation is limited to other Gram-negative species including *C*. *crescentus* and *P*. *aeruginosa*. We found that most transmembrane protein complexes follow this pattern. Interestingly, species with partial complex component conservation vs. *E*. *coli* may highlight situations in which core elements of a complex are conserved but have been modified to carry out other functions or adapted to special physiological circumstances. For example, 3 out of 4 of the succinate dehydrogenase complex (EcoCyc: SUCC-DEHASE) components in *E*. *coli* are also present in *B*. *subtilis* but not at all in *S*. *sanguinis*. This is an especially interesting example as two of the components, SdhC and SdhD, are inner membrane proteins, though only SdhC is present in the three-component *B*. *subtilis* succinate dehydrogenase. We conclude that membrane proteins and their complexes are particularly malleable, given their role in signaling and transport which reflects adaptations to specific environments and the nutrients present in them.

Smaller and more reduced bacterial genomes (that is, relative to *E*. *coli*) appear to code for a greater fraction of highly-conserved protein complexes. This conservation is evident in comparisons of the *Mycoplasma pneumoniae* protein complexes. In an examination of these protein complex components across more than 800 bacterial genomes, we found that species such as *M*. *pneumoniae* offers a better model of the protein complexes most critical to bacterial life. Protein complexes observed in *M*. *pneumoniae* may not only have retained a core set of functions but also utilized a higher degree of multifunctionality among its metabolic enzymes [18,19].

Surprisingly, many essential proteins are poorly conserved and essentiality itself is often not conserved across species (Figs. 4 through 6). This suggests that many functions can be replaced by non-homologous displacement [20] and that genomes are more malleable in evolutionary terms than previously expected. Clearly, this evolutionary flexibility has contributed much to the success of microbes to populate all possible environments on the planet. Variability in complex conservation highlights a limitation with this study: we are unavoidably limited by the availability of sequenced bacterial genomes. Newly-characterized genomes may reveal additional variation or consistency among protein complexes even if they are highly reduced in other respects.

As with their protein components, individual complexes reveal underlying evolutionary processes (Fig. 6 and S5 Fig.). The most highly-conserved complexes are those with functions critical to microbial life, including transcription, translation, and transcript degradation. Though different RNA polymerase (RNAP) holoenzymes (that is, RNA polymerases with different sigma factors) were considered as distinct complexes in this study, all bacterial species unsurprisingly retained at least one type of RNAP. The ribosome (EcoCyc: CPLX0–3964) is also well-conserved though its size and high level of conservation may obscure cross-species differences.

Variable conservation of some complexes is visible even among the *Escherichia* genomes. CPLX0–7909 (the RnlA-RnlB toxin-antitoxin complex) only appears to be present in K-12 *E*. *coli* but also in single species of *Shewanella* and *Photobacterium*. This toxin-antitoxin system has a role in bacteriophage resistance in *E*. *coli* [21] but it is unclear if this function may be retained in distantly related bacteria. CPLX0–2001 (the ferric dicitrate transport system) provides an example of more gradual change. This complex spans the membrane, suggesting its conservation should be membrane-dependent. This appears to be the case as it is well conserved across most Proteobacteria (except the Rickettsiales and *Buchnera* species) yet is poorly-conserved across most of the species traditionally considered Gram positive. A subset of complexes, including CPLX0–1163 (HslVU protease) and ABC-56-CPLX (aliphatic sulfonate ABC transporter), fit a strict co-conservation model: these complexes are almost always present in their full form rather than as a fraction of the *E*. *coli* model complex. These complexes are exceptions rather than the rule. Using *E*. *coli* as a model, few complexes are conserved perfectly across a wide range of species; in fact, most complexes are fractionally conserved.

## Materials and Methods

All data management was performed using in-house Python scripts (SPICEDNOG; available at http://github.com/caufieldjh/spicednog). Statistical analysis and clustering was performed using R package *vegan* [22].

### Data sources

The full set of protein complexes from *Escherichia coli* K-12 W3110 as defined by Hu et al. [5] was assigned membership in orthologous groups (OGs) from version 3 of the eggNOG database [10] such that each protein in a complex was assigned to a single OG. The remaining loci were referred to using their original locus identifiers (in this case, their b-codes) and were retained for all further analysis. The process was repeated for all protein complexes isolated by Kühner et al. [6] from *Mycoplasma pneumoniae* M129 and for *E*. *coli* protein complexes defined by the EcoCyc database [13]. A representative set of six other species (*Bacillus subtilis* 168, *Caulobacter crescentus*, *Helicobacter pylori* 26695, *Mycoplasma genitalium* G37, *Pseudomonas aeruginosa* UCBPP-PA14, and *Streptococcus sanguinis* SK36) for which whole-genome gene essentiality data was selected for in-depth analysis. This species set is referred to as the focused set. Lists of all protein-coding loci for each species were obtained using the respective full proteome sets from UniProt (see S4 Table for taxonomy IDs corresponding to all genomes used). Essentiality data was collected from the Database of Essential Genes [11]. Protein structures were obtained from the Protein Data Bank (www.rcsb.org, [23]) and are referenced where used.

A set of 894 species, referred to as the large set, was also prepared using every bacterial species present in eggNOG v.3 and in the NCBI Taxonomy database [24]. The trees shown in Figs. 4, 5, and 6 are cladograms intended to show the general relationship between species within context of consensus taxonomy.

### Orthologous groups

Each locus in each genome was assigned to a single orthologous group (OG) as in eggNOG v.3 [10], such that all loci were assigned to a COG, a NOG, or a bactNOG, depending upon the most widely-conserved group assignment available (see Powell et al. [10] for details regarding OG levels). Next, the presence of each locus was determined across the entire set of bacterial species; a locus seen in half of all bacterial species would be assigned a conservation value of 0.5. This presence was averaged across all loci to generate a value for average locus conservation for each genome. This value was adjusted based on locus coverage in eggNOG (i.e., if only 70 percent of the loci in a genome mapped to eggNOG OGs, the average value was reduced by 30 percent.) An identical set of comparisons were performed for all loci with predicted paralogs (that is, loci with the same OG assignment) removed prior to comparison. Subsets of selected species were also prepared such that they included only loci with the same orthologous groups as those seen in the Hu et al., EcoCyc, or Kühner et al. protein complex sets. Genome sizes were retrieved from NCBI GenBank and KEGG GENOME [25].

### Comparing complex composition to a random model to observe the distribution of essentiality

The observed distributions of essential genes among those coding for protein complex components were obtained using protein complex sets [5,6,13], eggNOG (v. 3) [10], and the Database of Essential Genes [11] as defined above. For a single protein complex, an essentiality fraction was defined as the fraction of all genes in a complex found to be essential, out of the set of all unique protein-coding genes in the complex. The conservation scores were used to judge participation of a complex within a dataset, establishing a maximum for each species and dataset combination. Second, essentiality fraction was found by linking each essential protein to an OG. In instances where multiple proteins shared OGs but not essentiality, essentiality was considered as the primary case and the OG was counted as essential.

A random model was created for the purpose of comparing the data set to background noise. The random model, meant to represent a collection of randomly sized complexes, was populated by proteins that have been randomly assigned essential status. The complex sizes were randomly assigned a value from three to ten. Each complex was then assigned protein values of either essential or non-essential status. The probability of being essential was determined by the overall percent of essential genes within the organism, while the random model size is equal to the maximum of the species and dataset being compared. This random model was then put through the same binning process as the observed data. The mean of each bin was obtained after 10,000 replications. This results in s bins of a size that is no longer equal to the actual data set but demonstrates an appropriate background noise level for comparison purposes. The log_2_(Observed/Expected) values are plotted in Fig. 8 to show any significant difference between observed essentiality and expected.

### Comparative proteome and complexome analysis

The general scheme for data analysis was as follows: (1) A list of all orthologous groups (OG) was produced for each of 894 bacterial species found in the large set defined above. (2) Presence or absence of each OG was determined for all species. (3) Repeated OGs were removed from each list and step 2 was repeated. (4) The list from step 1 was used to map OGs to the components of three sets of protein complexes. The complexes were compared to search for cross-data set complex matches. Gene essentiality was also mapped to each OG in a species-dependent basis. (5) A list of 8 taxonomically-divergent species was selected and used to define fractional conservation and fractional essentiality of each protein complex.

OGs were used as the basis of comparison for similarity between data sets. **Complex size** was defined as the number of unique proteins isolated from a complex; i.e. a complex may contain 3 unique OGs but 4 distinct protein components, yielding a complex size of 4. For each complex, the presence of each OG within the complex was assayed in the full proteome sets of the seven other representative species. The resulting binary presence/absence values were combined to produce a value for the percent complex conservation. This value intentionally disregards any gene context similarity (that is, an OG may be present in two genomes even if neighboring genes differ between the genomes) and simply expresses the fraction of complex components which a specific genome may code for. When a target proteome did contain a specified complex component, the number of paralogs of the component-coding gene was determined as the number of proteins in the list mapping to the same OG. While further verification, may be necessary to define any of these protein-coding genes as true paralogs, we simply used the OGs (including paralogs) as determined by eggNOG.

All protein complex components were also assigned binary **essentiality** values using published assays specific to the species listed above. These values were used to define the essentiality fraction of each potentially conserved complex, i.e. an *E*. *coli* complex for which 80% of the components appear to be conserved in *M*. *pneumoniae* but only 60% of the components may be essential in the latter species.

A broader comparison was prepared using the list of 894 species as defined above. Genome sizes for each species were retrieved from the KEGG GENOME Database (http://www.genome.jp/kegg/genome.html, [25]). For each species, the total number of OG-mapped protein-coding loci was divided by the total number of loci to produce a value for percentage mapped. Using the list of all OGs in the species, each OG was compared with all other species to determine its conservation across Bacteria. Adjusted **average locus conservation** for a particular genome, *C*_*AAL*_ *(g)*, was calculated as:

$$C_{AAL}(g)=m\frac{\left(\frac{\sum C_{L}(g)}{L(g)}\right)}{N}$$

where *C*_*L*_ is the number of genomes in which the locus is present, *L(g)* is the number of loci in the genome, N is the total number of genomes, and m is the percentage of loci mapped by eggNOG v.3. Values are adjusted using the fraction of loci actually mapped so unmapped loci lower the effective conservation.

An identical list of values, but with repeated OGs reduced to a single occurrence, was averaged to produce average OG conservation. This modification removes the effect of counting loci more than once when they share OGs, as may happen when two or more loci are paralogous. Adjusted **average OG conservation** for a particular genome, *C*_*AAO*_ *(g)*, was calculated as:

$$C_{AAO}(g)=m\frac{\left(\frac{\sum C_{L}(g)}{O(g)}\right)}{N}$$

where *C*_*L*_ is the number of genomes in which the locus is present, *O(g)* is the number of unique OGs in the genome, N is the total number of genomes, and m is the percentage of loci mapped by eggNOG v.3.

Species/strains were sorted by genome size and compared to the average conservation values. For the set of all bacterial genomes, *N* = 943, though Fig. 1 presents the results after removing 45 genomes of very similar size and sequence. For a subset of species, the Average Locus and Average OG Conservation values were calculated using only OGs found in published protein complex data sets.

Mapping of fractional complex conservation across species was performed as follows for both the focused set (8 species) and the large set. A cladogram of all species in the set was prepared using the Interactive Tree of Life (iTOL, [26]) project as per NCBI taxonomy. All protein components were mapped to eggNOG v.3 OGs and complex size was determined as defined above. Conservation fraction of each complex in each species was defined as the number of complex component OGs shared between the model (an *E*. *coli* complex) and the target genome over the size of the model complex. Heatmaps were prepared using the R *heatmap*.*2* function in the *gplots* package. Randomized models of the large set heatmaps (Fig. 5, S5 and S6 Figs.) retaining the same species order but with a randomized distribution of conservation fractions were prepared using the R function *randomizeMatrix* (in the *picante* package [27]) and the ‘richness’ null model to respect overall conservation levels.

## Supporting Information

S1 Fig All EcoCyc complexes and their fractional conservation in selected bacterial species. An extended version of Fig. 5. Names with blue stars indicate example complexes shown in Fig. 4.
(PDF)

S2 Fig All EcoCyc complexes and their fractional essentiality in selected bacterial species. An extended version of Fig. 5. Names with blue stars indicate example complexes shown in Fig. 4.
(PDF)

S3 Fig Cross-species conservation of experimentally-observed protein complexes and the sums of the counts of potential paralogs of their components. **(A)** Proteins in *E*. *coli* complexes [5] tend to have more paralogs if the complexes are less conserved. **(B)** By contrast, in *M*. *pneumoniae* complexes [6] more conserved complexes tend to have more paralogous proteins. Fraction of conservation and sum of paralogy are calculated as described in Materials and Methods. Each node represents a single protein complex with relative size corresponding to the size of the complex in number of components. *E*. *coli* complexes as defined by Hu et al. were compared to the full *M*. *pneumoniae* proteome while *M*. *pneumoniae* complexes were compared to the full *E*. *coli* proteome; all cross-species comparison are done using predicted orthologs as described in Materials and Methods.
(PDF)

S4 Fig Essentiality of protein complexes and their average paralogy. More essential protein complexes tend to have fewer components with paralogs, at least in *E*. *coli* literature-curated Ecocyc **(A)** and experimentally-observed Hu et al. **(B)** complexes. However, this is not true in reduced genomes such as that of *Mycoplasma pneumoniae* **(C)**. Each node represents a single protein complex with relative size corresponding to the size of the complex in number of components. Fraction of essentiality and average paralogy are calculated as described in Materials and Methods. Data from Hu et al. [5] (A), EcoCyc [13] (B) and [6] (C). **(D)** An example complex from each of the three data sets is shown. These complexes are not identical in composition but have similar components. Each complex, as defined by a single data set, may offer an incomplete set of protein components and may overlook the impact of paralogy.
(PDF)

S5 Fig *E*. *coli* literature-curated complex conservation across bacteria corresponds to taxonomic boundaries. This figure expands upon that in Fig. 6. Using the EcoCyc set of protein complexes as a model, each column is a single complex from the set and each row is a distinct bacterial genome. 285 complexes and 894 genomes are shown in total. Genome order corresponds to a cladogram produced using NCBI taxonomy and an Interactive Tree of Life (iTOL, [26]) tree. Color gradients correspond to fractional conservation. Complexes (columns) have been clustered on the basis of the distance between their average fractional conservations (average linkage).
(PDF)

S6 Fig *E*. *coli* experimentally-observed complex conservation across bacteria corresponds to taxonomic boundaries. Using the Hu et al. set of protein complexes as a model, each column is a single complex from the set and each row is a distinct bacterial genome. 310 complexes and 894 genomes are shown in total. Genome order corresponds to a cladogram produced using NCBI taxonomy and an Interactive Tree of Life (iTOL, [26]) tree. Color gradients correspond to fractional conservation. Complexes (columns) have been clustered on the basis of the distance between their average fractional conservations (average linkage).
(PDF)

S1 Text Guide to content provided in the supporting tables. (DOCX)

S1 Table Key to protein complex IDs as shown in Fig. 5. (XLSX)

S2 Table Protein complex conservation across bacteria. (XLSX)

S3 Table Conservation of orthologous groups between species pairs. (XLSX)

S4 Table Conservation across numerous bacterial species. (XLSX)

S5 Table Conservation of *E*. *coli* complexes from Hu et al. (2009). (XLSX)

S6 Table Essentiality of *E*. *coli* complexes from Hu et al. (2009). (XLSX)

S7 Table Conservation of *E*. *coli* complexes from EcoCyc. (XLSX)

S8 Table Essentiality of *E*. *coli* complexes from EcoCyc. (XLSX)

S9 Table Conservation of *Mycoplasma pneumoniae* complexes from Kühner et al. (2009). (XLSX)

S10 Table Essentiality of *Mycoplasma pneumoniae* complexes from Kühner et al. (2009). (XLSX)

S11 Table Experimental protein complexes containing uncharacterized components. (XLSX)

## References

[1] Ryan CJ, Roguev A, Patrick K, Xu J, Jahari H, Tong Z, et al Hierarchical modularity and the evolution of genetic interactomes across species. Molecular Cell. 2012;46(5):691–704. 10.1016/j.molcel.2012.05.028 22681890 PMC3380636

[2] Dixon SJ, Costanzo M, Baryshnikova A, Andrews B, Boone C. Systematic mapping of genetic interaction networks. Annual Review of Genetics. 2009;43:601–25. 10.1146/annurev.genet.39.073003.114751 19712041

[3] Ryan CJ, Krogan NJ, Cunningham P, Cagney G. All or nothing: protein complexes flip essentiality between distantly related eukaryotes. Genome Biology and Evolution. 2013;5(6):1049–59. 10.1093/gbe/evt074 23661563 PMC3698920

[4] Arifuzzaman M, Maeda M, Itoh A, Nishikata K, Takita C, Saito R, et al Large-scale identification of protein-protein interaction of Escherichia coli K-12. Genome Research. 2006;16:686–91. 16606699 10.1101/gr.4527806 PMC1457052

[5] Hu P, Janga SC, Babu M, Díaz-Mejía JJ, Butland G, Yang W, et al Global functional atlas of Escherichia coli encompassing previously uncharacterized proteins. PLoS Biology. 2009;7:e96 10.1371/journal.pbio.1000096 19402753 PMC2672614

[6] Kühner S, van Noort V, Betts MJ, Leo-Macias A, Batisse C, Rode M, et al Proteome organization in a genome-reduced bacterium. Science (New York, NY). 2009;326:1235–40. 10.1126/science.1176343 19965468

[7] Sharan R, Ideker T, Kelley B, Shamir R, Karp RM. Identification of protein complexes by comparative analysis of yeast and bacterial protein interaction data. Journal of Computational Biology: a journal of computational molecular cell biology. 2005;12:835–46. 16108720 10.1089/cmb.2005.12.835

[8] de Matos Simoes R, Dehmer M, Emmert-Streib F. Interfacing cellular networks of S. cerevisiae and E. coli: connecting dynamic and genetic information. BMC genomics. 2013;14:324 10.1186/1471-2164-14-324 23663484 PMC3698017

[9] Cohen O, Ashkenazy H, Levy Karin E, Burstein D, Pupko T. CoPAP: Coevolution of presence-absence patterns. Nucleic acids research. 2013;41(Web Server issue):W232–7. 10.1093/nar/gkt471 PMC3692100 23748951

[10] Powell S, Szklarczyk D, Trachana K, Roth A, Kuhn M, Muller J, et al eggNOG v3.0: orthologous groups covering 1133 organisms at 41 different taxonomic ranges. Nucleic acids research. 2012;40:D284–9. 10.1093/nar/gkr1060 22096231 PMC3245133

[11] Luo H, Lin Y, Gao F, Zhang CT, Zhang R. DEG 10, an update of the database of essential genes that includes both protein-coding genes and noncoding genomic elements. Nucleic acids research. 2014;42(Database issue):D574–80. 10.1093/nar/gkt1131 24243843 PMC3965060

[12] McCutcheon JP, McDonald BR, Moran NA. Origin of an alternative genetic code in the extremely small and GC-rich genome of a bacterial symbiont. PLoS genetics. 2009;5(7):e1000565 10.1371/journal.pgen.1000565 19609354 PMC2704378

[13] Keseler IM, Mackie A, Peralta-Gil M, Santos-Zavaleta A, Gama-Castro S, Bonavides-Martínez C, et al EcoCyc: fusing model organism databases with systems biology. Nucleic acids research. 2013;41:D605–12. 10.1093/nar/gks1027 23143106 PMC3531154

[14] Liechti G, Goldberg JB. Outer membrane biogenesis in Escherichia coli, Neisseria meningitidis, and Helicobacter pylori: paradigm deviations in H. pylori. Frontiers in cellular and infection microbiology. 2012;2:29 10.3389/fcimb.2012.00029 22919621 PMC3417575

[15] Eraso JM, Markillie LM, Mitchell HD, Taylor RC, Orr G, Margolin W. The highly conserved MraZ protein is a transcriptional regulator in Escherichia coli. Journal of bacteriology. 2014;196(11):2053–66. 10.1128/JB.01370-13 24659771 PMC4010979

[16] Hart GT, Lee I, Marcotte ER. A high-accuracy consensus map of yeast protein complexes reveals modular nature of gene essentiality. BMC bioinformatics. 2007;8:236 17605818 10.1186/1471-2105-8-236 PMC1940025

[17] Wang PI, Marcotte EM. It’s the machine that matters: Predicting gene function and phenotype from protein networks. Journal of proteomics. 2010;73(11):2277–89. 10.1016/j.jprot.2010.07.005 20637909 PMC2953423

[18] Yus E, Maier T, Michalodimitrakis K, van Noort V, Yamada T, Chen W-H, et al Impact of genome reduction on bacterial metabolism and its regulation. Science (New York, NY). 2009;326:1263–8. 10.1126/science.1177263 19965476

[19] Kelkar YD, Ochman H. Genome reduction promotes increase in protein functional complexity in bacteria. Genetics. 2013;193:303–7. 10.1534/genetics.112.145656 23114380 PMC3527252

[20] Koonin EV, Mushegian AR, Bork P. Non-orthologous gene displacement. Trends in genetics: TIG. 1996;12(9):334–6. 8855656

[21] Wei Y, Gao ZQ, Otsuka Y, Naka K, Yonesaki T, Zhang H, et al Structure-function studies of Escherichia coli RnlA reveal a novel toxin structure involved in bacteriophage resistance. Molecular microbiology. 2013;90(5):956–65. 10.1111/mmi.12409 24112600

[22] Oksanen J, Blanchet F, Kindt R, Legendre P, Minchin P, O’Hara R, et al vegan: Community Ecology Package. R package version 2.0–7. 2013.

[23] Rose PW, Bi C, Bluhm WF, Christie CH, Dimitropoulos D, Dutta S, et al The RCSB Protein Data Bank: new resources for research and education. Nucleic acids research. 2013;41(Database issue):D475–82. 10.1093/nar/gks1200 23193259 PMC3531086

[24] Federhen S. The NCBI Taxonomy database. Nucleic acids research. 2012;40(Database issue):D136–43. 10.1093/nar/gkr1178 22139910 PMC3245000

[25] Kanehisa M, Goto S. KEGG: kyoto encyclopedia of genes and genomes. Nucleic acids research. 2000;28:27–30. 10592173 10.1093/nar/28.1.27 PMC102409

[26] Letunic I, Bork P. Interactive Tree Of Life v2: online annotation and display of phylogenetic trees made easy. Nucleic acids research. 2011;39(Web Server issue):W475–8. 10.1093/nar/gkr201 21470960 PMC3125724

[27] Kembel SW, Cowan PD, Helmus MR, Cornwell WK, Morlon H, Ackerly DD, et al Picante: R tools for integrating phylogenies and ecology. Bioinformatics. 2010;26(11):1463–4. 10.1093/bioinformatics/btq166 20395285

