# Supplementary material for: Protein Complexes in Bacteria
Source: PLoS Comput Biol. 2015 Feb 27;11(2):e1004107. doi: 10.1371/journal.pcbi.1004107 (PMC4344305; doi:10.1371/journal.pcbi.1004107)
Supplement: S1 Fig — An extended version of Fig. 5. Names with blue stars indicate example complexes shown in Fig. 4. (PDF) [file pcbi.1004107.s001.pdf]

Figure S1

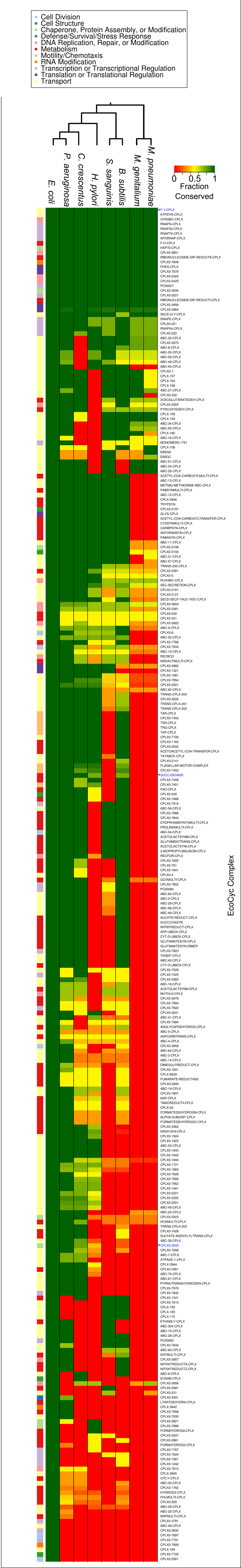

Cell Division
Cell Structure
Chaperone, Protein Assembly, or Modification
Defense/Survival/Stress Response
DNA Replication, Repair, or Modification
Metabolism
Motility/Chemotaxis
RNA Modification
Transcription or Transcriptional Regulation
Translation or Translational Regulation
Transport
E. coli
P. aeruginosa
C. crescentus
H. pylori
S. sanguinis
B. subtilis
M. genitalium
M. pneumoniae
0
0.5
1
Fraction Conserved
EcoCyc Complex
