# Supplementary figures and images for: Protein Complexes in Bacteria
Source: PLoS Comput Biol. 2015 Feb 27;11(2):e1004107. doi: 10.1371/journal.pcbi.1004107 (PMC4344305; doi:10.1371/journal.pcbi.1004107)

Figure S2

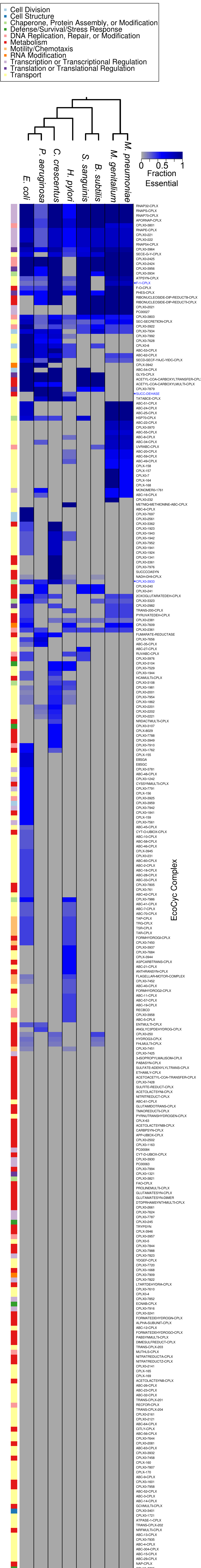

Supplement: S2 Fig — An extended version of Fig. 5. Names with blue stars indicate example complexes shown in Fig. 4. (PDF) [file pcbi.1004107.s002.pdf]

Figure S3

A

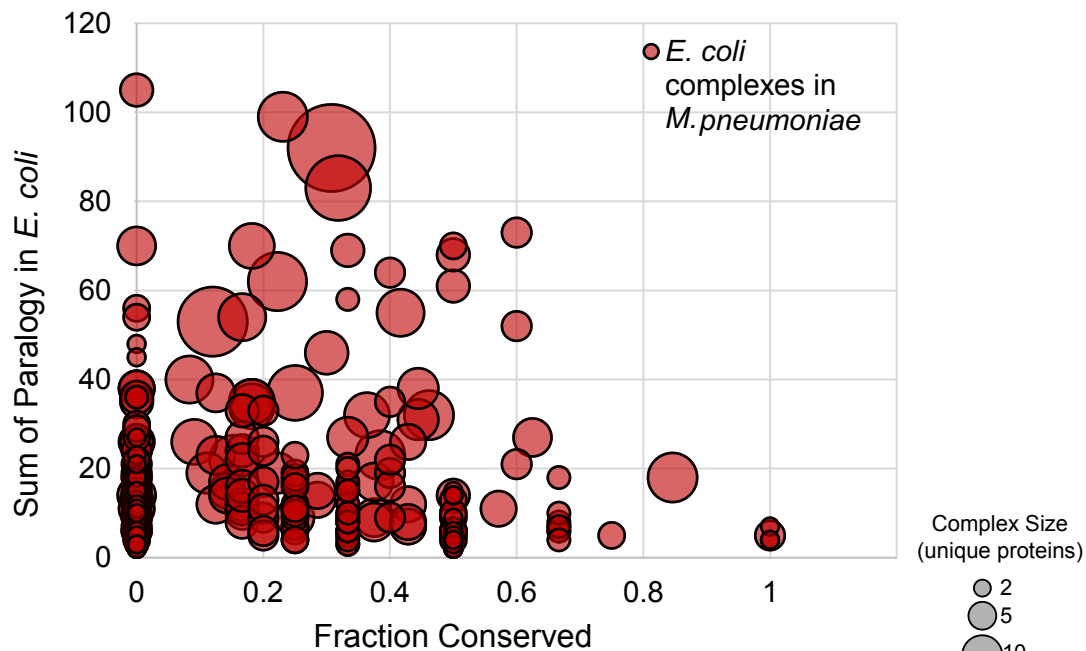

B

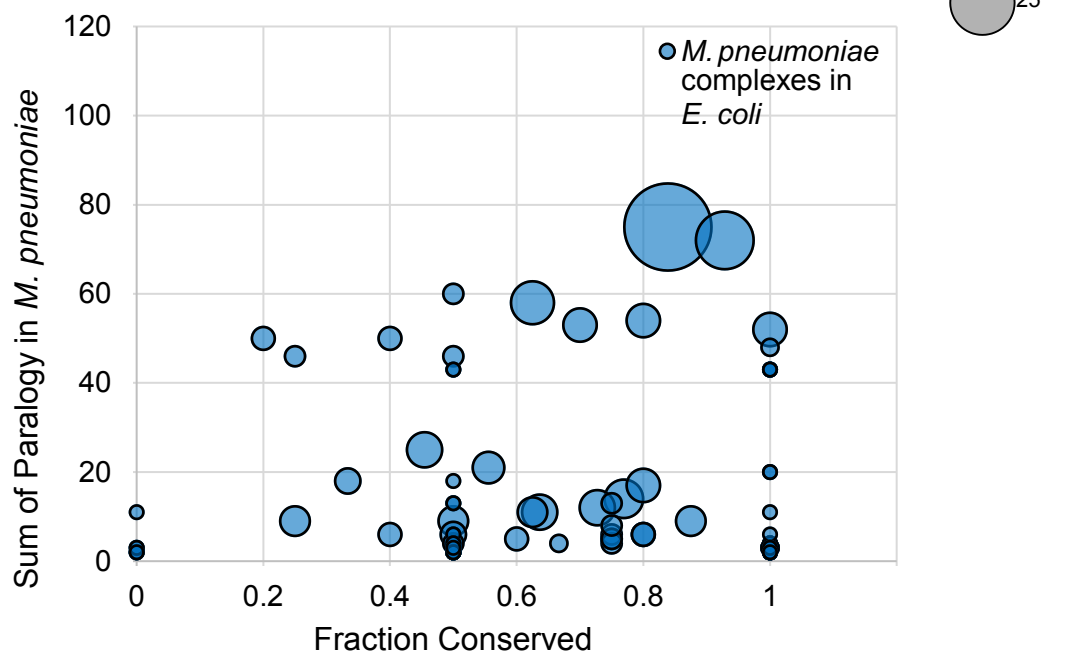

Supplement: S3 Fig — (A) Proteins in E. coli complexes [5] tend to have more paralogs if the complexes are less conserved. (B) By contrast, in M. pneumoniae complexes [6] more conserved complexes tend to have more paralogous proteins. Fraction of conservation and sum of paralogy are calculated as described in Materials and Methods. Each node represents a single protein complex with relative size corresponding to the size of the complex in number of components. E. coli complexes as defined by Hu et al. were compared to the full M. pneumoniae proteome while M. pneumoniae complexes were compared to the full E. coli proteome; all cross-species comparison are done using predicted orthologs as described in Materials and Methods. (PDF) [file pcbi.1004107.s003.pdf]

Figure S4

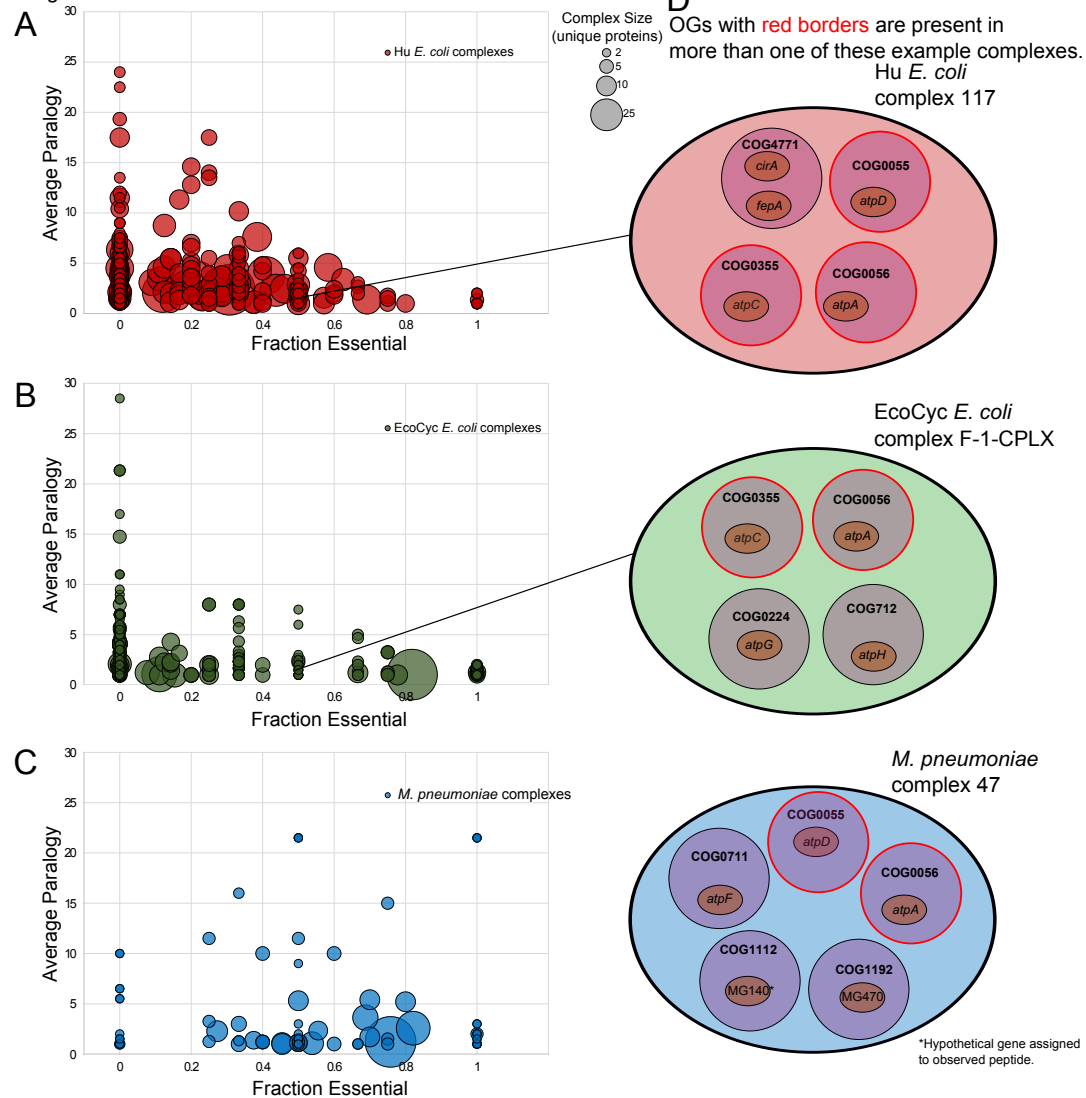

Supplement: S4 Fig — More essential protein complexes tend to have fewer components with paralogs, at least in E. coli literature-curated Ecocyc (A) and experimentally-observed Hu et al. (B) complexes. However, this is not true in reduced genomes such as that of Mycoplasma pneumoniae (C). Each node represents a single protein complex with relative size corresponding to the size of the complex in number of components. Fraction of essentiality and average paralogy are calculated as described in Materials and Methods. Data from Hu et al. [5] (A), EcoCyc [13] (B) and [6] (C). (D) An example complex from each of the three data sets is shown. These complexes are not identical in composition but have similar components. Each complex, as defined by a single data set, may offer an incomplete set of protein components and may overlook the impact of paralogy. (PDF) [file pcbi.1004107.s004.pdf]
