# Supplementary material for: Protein Complexes in Bacteria
Source: PLoS Comput Biol. 2015 Feb 27;11(2):e1004107. doi: 10.1371/journal.pcbi.1004107 (PMC4344305; doi:10.1371/journal.pcbi.1004107)
Supplement: S1 Text — (DOCX) [file pcbi.1004107.s007.docx]

**Text S1**

**Supplementary Information for Protein Complexes in Bacteria**

Caufield JH, Abreu M, Wimble C, and Uetz P

See Materials and Methods for further detail regarding data sources.

In these tables and in the manuscript, counts of orthologous groups are treated in two distinct ways.

All orthology assignments are applied using orthologous groups (OGs) from eggNOG v.3 (Powell et al., 2012; http://eggnog.embl.de/version_3.0/ ).

COGs were used by default. For proteins not mapped to COGs, NOGs or bactNOGs were used. See Powell et al. for descriptions of these orthologous group types.

Loci counts are those in which each locus has been mapped to an OG for comparison across species. When more than one locus maps to the same OG, all are included.

OG counts are those in which each locus has been mapped to an OG and multiple OG members are combined. When more than one locus maps to the same OG, all are treated as a single OG.

Essentiality data for *M. pneumoniae* is predicted based on that for *M. genitalium*; see Glass et al. (2006) PNAS.

Species sets used in this study are either the focused set (containing the 8 species below) or a large set (as in Table S2).

The focused set contains:

*Mycoplasma genitalium* G37

*Mycoplasma pneumoniae* M129

*Streptococcus sanguinis* SK36

*Caulobacter crescentus* NA1000

*Bacillus subtilis* subsp. *subtilis* 168

*Escherichia coli* K-12 W3110

*Pseudomonas aeruginosa* UCBPP-PA14

**Table Contents**

S1 Key to protein complex IDs.

As used in Figure 5 in the manuscript.

Each complex has an identifier and common name originally assigned by EcoCyc (Keseler et al., 2013).

S2 Protein complex conservation across Bacteria.

These tables define the large set of species used in this study.

Species and strains (first column) are identified by their NCBI Taxonomy ID and are arranged as per their location in a cladogram prepared using the interactive Tree Of Life project (Letunic and Bork 2011 Nucleic Acids Res).

Table S2A contains values for presence or absence of protein complex component conservation across Bacteria.

Each column corresponds to a single OG (as used in eggNOG v.3) corresponding a protein found in at least one EcoCyc *E. coli* K-12 protein complex

Values indicate presence or absence of the OG in the target species; a value of 1 indicates the OG is found at least once in the target genome.

Table S2B contains values for fractional protein complex conservation across Bacteria.

These values are those presented in the heatmap in Figure 6.

These data include fraction of conservation relative to *E. coli* K-12 for 285 EcoCyc complexes across 894 genomes in total.

Fraction of conservation is the fraction of unique protein components in the reference species and strain (E. coli K-12 MG1655) present in the target species and strain's genome as orthologs.

A value of 1 indicates orthologs of all reference complex components appear to be present in the target species.

A value of 0 indicates no orthologs of any reference complex components appear to be present in the target species.

Conservation is based on presence/absence - paralogy is not considered in this context.

Protein complexes are identified using their EcoCyc IDs.

S3 Conservation of orthologous groups between species pairs.

Table S3A shows the percentages of loci shared between two of eight species, along with the total number of loci for that species, where each locus has been mapped to an OG.

Table S3B shows the counts of loci that are shared between two species.

Tables S3C and S3D are as in tables A and B, respectively, but with multiple members of each OG removed.

S4 Conservation across numerous bacterial species.

See Figure 1.

Species is the name of a single bacterial species, strain, or substrain where applicable.

Species Code is the unique species identifier used by Uniprot and eggNOG.

Genome Size is the size of the genome of this species in megabases. This value includes all genome regions and, where applicable, plasmids characteristic of the strain.

Loci refers to the total number of protein-coding loci present in the genome as reported by eggNOG v.3.

Mapped Loci refers to the total number of protein-coding loci assigned any level of OG by eggNOG v.3.

% Mapped is the total percentage of protein-coding loci in the specified genome with any level of OG assignment in eggNOG v.3.

Values are shaded from blue to red to indicate high to low mapping, respectively.

Average Locus Conservation is calculated as follows: each protein-coding locus is assigned an OG, the presence or absence of which is determined for every other genome in the data set.

The percentage of genomes in the data set containing the OG at least once is used as Locus Conservation. All values are averaged together for a single genome to produce a value for Average Locus Conservation.

Adjusted Average Locus Conservation is Average Locus Conservation reduced by the fraction of un-mapped protein-coding loci.

This adjustment assumes loci are un-mapped due to lack of conservation beyond one genome.

Values are shaded from blue to red to indicate high to low conservation, respectively.

Average OG Conservation is calculated in an identical manner as Average Locus Conservation, but multiple members of a single OG (as is the case with paralogs) are reduced to a single OG per genome.

Adjusted Average OG Conservation is calculated in an identical manner as Adjusted Average Locus Conservation, with the conservation percentage value reduced by the fraction of un-mapped protein-coding loci.

Values are shaded from blue to red to indicate high to low conservation, respectively.

Average conservation - Difference between locus and OG refers to the difference in percentage values between Average Locus Conservation and Average OG Conservation.

These values are rough estimates of the effect of paralogy on average conservation values; higher values indicate a greater effect.

Some difference values, especially among genomes < 1 Mb, are negative. This may indicate very low paralogy effects and/or high conservation of single, non-paralogous genes.

Average OG conservation among complex components is calculated as Average OG Conservation is, but with the OGs present in a specific set of protein complexes.

The three protein complexes used in this study were used to define the OG subsets.

The presence/absence of each OG in each subset was determined for each of the 8 bacterial species focused on in this study.

Average OG Conservation was calculated as above for the subset of OGs present in each of the 8 species.

Tables S5 through S10 compare individial protein components of complexes. They share the following variables:

These tables use the focused set of species as defined above.

Complex ID is the complex identifier assigned by the data source. For the experimentally-observed complexes (Hu et al. (2009) and Kühner et al (2009)) this is the CplxID or Purification ID, respectively.

For the literature-curated EcoCyc complexes, Complex IDs are the unique identifiers used within the database.

This column also includes the names of the eight species focused on in this study. All values in the row apply to the named species.

Complex Members in Original Complex is the size of the complex in number of unique proteins. An OG may be present more than once in a complex - this does not change the value.

Conservation fraction / Essentiality fraction are the fractions of protein components conserved in the species or essential in the species, respectively, out of all components present in the originally defined complex.

Essentiality fraction is not the fraction of conserved essentiality, but rather all genes in the species observed to be conserved and essential out of all components present in the original complex.

A conservation fraction of 1 indicates all components of the original complex appear to be conserved in the genome of the named species.

An essentiality fraction of 1 indicates all components of the original complex appear to be present and essential in the named species.

In most cases, essentiality fraction is <1, even for the original complex. This is expected as not every component of a complex may be essential, even when the complex itself is essential.

S5 Conservation of E. coli complexes from Hu et al. (2009).

See Table S7 in Hu et al (2009) for the original complex list.

Values indicate number of OG members present in a respective genome. Red is no presence, yellow is a single occurence, green indicates possible paralogy.

S6 Essentiality of E. coli complexes from Hu et al. (2009).

Complexes are the same as in Table S4.

Values indicate essentiality (green), no evidence of essentiality (red), or gene not present (blank).

S7 Conservation of E. coli complexes from EcoCyc.

The complex list includes all protein complexes identified by the database with the exception of homomers.

Values indicate number of OG members present in a respective genome. Red is no presence, yellow is a single occurence, green indicates possible paralogy.

S8 Essentiality of E. coli complexes from EcoCyc.

Complexes are the same as in Table S6.

Values indicate essentiality (green), no evidence of essentiality (red), or gene not present (blank).

S9 Conservation of Mycoplasma pneumoniae complexes from Kühner et al (2009).

See Table S2 in Kühner et al (2009) for the original complex list.

Values indicate number of OG members present in a respective genome. Red is no presence, yellow is a single occurence, green indicates possible paralogy.

S10 Essentiality of Mycoplasma pneumoniae complexes from Kühner et al (2009).

Complexes are the same as in Table S8.

Values indicate essentiality (green), no evidence of essentiality (red), or gene not present (blank).

S11 Experimental protein complexes containing uncharacterized components.

Data Set identifies the source of the complex.

Complex ID is the complex identifier assigned by the data source. For the experimentally-observed complexes (Hu et al. (2009) and Kühner et al (2009)) this is the CplxID or Purification ID, respectively.

Size is the number of unique protein components (by OG) in the complex.

Unknown Components is the total number of components in this complex with unknown or unclear functions beyond their membership in the complex.

The corresponding OGs must have functional category labels of R or S.

Highly Conserved Components are those coded for by the genomes of at least half of the eight species in the focused set.

Highly Essential Components are those coded for by essential genes present in at least half of the eight species in the focused set.
