# Supplementary material for: Protein Complexes in Bacteria
Source: PLoS Comput Biol. 2015 Feb 27;11(2):e1004107. doi: 10.1371/journal.pcbi.1004107 (PMC4344305; doi:10.1371/journal.pcbi.1004107)
Supplement: S5 Fig — This figure expands upon that in Fig. 6. Using the EcoCyc set of protein complexes as a model, each column is a single complex from the set and each row is a distinct bacterial genome. 285 complexes and 894 genomes are shown in total. Genome order corresponds to a cladogram produced using NCBI taxonomy and an Interactive Tree of Life (iTOL, [26]) tree. Color gradients correspond to fractional conservation. Complexes (columns) have been clustered on the basis of the distance between their average fractional conservations (average linkage). (PDF) [file pcbi.1004107.s005.pdf]

Figure S5

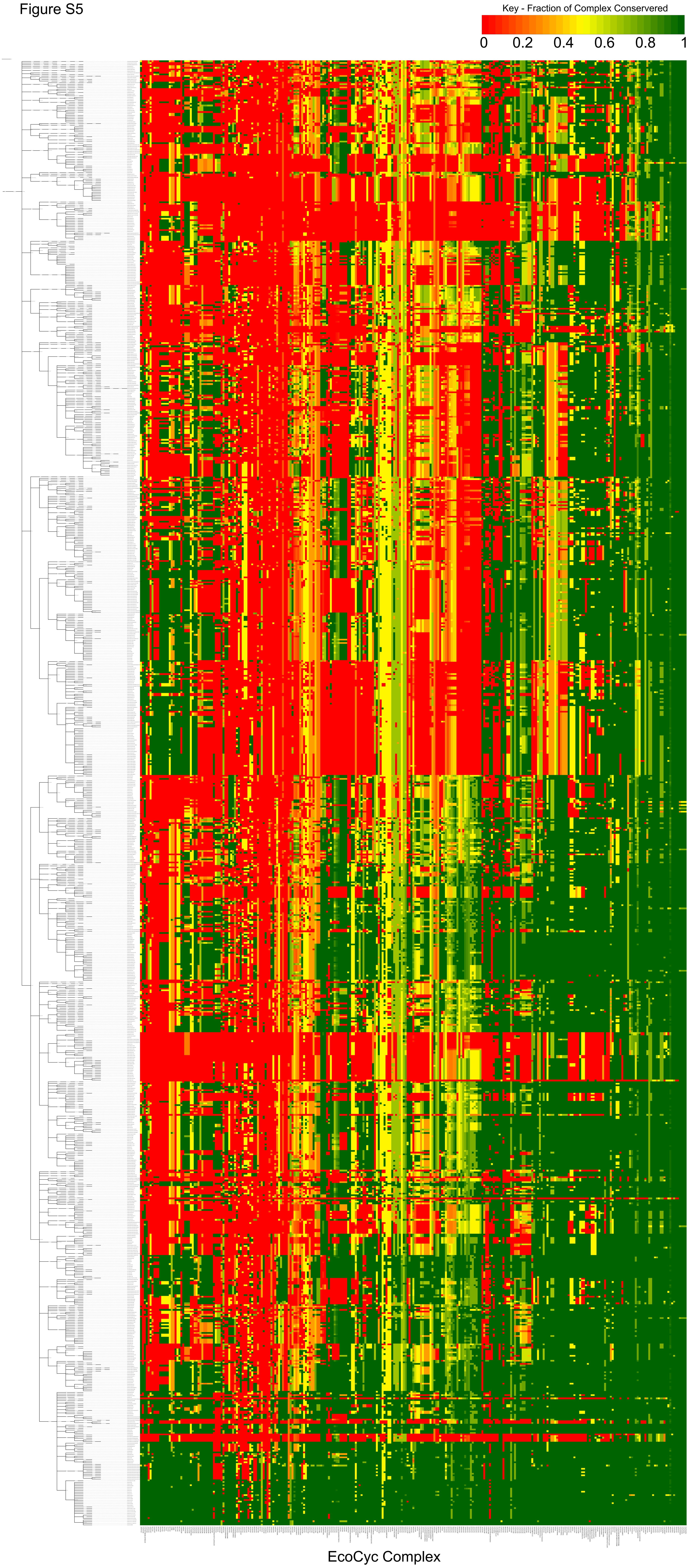

Key - Fraction of Complex Conserved
0
0.2
0.4
0.6
0.8
1
EcoCyc Complex
